# Computational optical streak microscopy of megahertz acoustic microbubble dynamics

**DOI:** 10.1186/s43074-026-00232-8

**Published:** 2026-05-01

**Authors:** Miguel Marquez, Yingming Lai, Miao Liu, Elahe Memari, Brandon Helfield, Jinyang Liang

**Affiliations:** 1 Laboratory of Applied Computational Imaging, Centre Énergie Matériaux Télécommunications, Institut National de la Recherche Scientifique, Université du Québec, 1650 Boulevard Lionel-Boulet, Varennes, Québec J3X1P7 Canada; 2 Department of Physics, Concordia University, 7141 Sherbrooke St. W., Montreal, Québec H4B1R6 Canada; 3 Department of Biology, Concordia University, 7141 Sherbrooke St. W., Montreal, Québec H4B1R6 Canada

**Keywords:** Ultrahigh-speed microscopy, Computational imaging, Deep learning, Acoustic microbubble dynamics, Microbubble-cell interactions

## Abstract

Real-time dynamic imaging of microbubbles is crucial for understanding their microscale biophysical interactions and advancing ultrasound therapy. Despite progress in time-resolved optical imaging, existing techniques still face trade-offs between acquisition speed, spatial resolution, affordability, and system complexity. Here, we introduce compressed optical-streaking dark-field ultrahigh-speed microscopy (COSDUM), a compact imaging platform that synergistically combines compressed sensing, streak imaging, dark-field microscopy, and deep learning. COSDUM compressively records megahertz acoustic microbubble dynamics over a wide field of view in a snapshot and reconstructs spatially resolved dynamics using a convolutional neural network-based algorithm. Using COSDUM, we captured stable cavitation, nonlinear oscillations, post-excitation free oscillations, and inertial collapse across microbubbles whose radii range from 0.5 to 2.1 μm. Applying COSDUM to microbubble-cell interaction in whole blood, we observed, for the first time, interplay between vibrating microbubbles and blood cells, including microbubble-driven platelet dynamics and highly asymmetric microbubble deformation and conformation around an adjacent red blood cell.

**Supplementary Information:**

The online version contains supplementary material available at 10.1186/s43074-026-00232-8.

## Introduction

Ultrasound-responsive microbubbles represent a promising strategy for targeted drug delivery. These microbubbles can be actuated with high spatial precision to transiently increase the permeability of biological barriers and enhance localized drug release, thereby improving therapeutic precision and minimizing systemic side effects [1–4]. Among these agents, encapsulated perfluoropropane microbubbles, stabilized by phospholipid or polymer shells, have shown strong potential in preclinical and clinical contexts [5–7]. Upon acoustic excitation, these gas-filled microspheres undergo volumetric oscillations that produce localized mechanical forces, including shear stress [8, 9], microstreaming [10, 11], and transient pore formation [12, 13]. These effects can temporarily disrupt cell membranes or modulate vascular permeability, enabling therapeutic agents to cross otherwise restrictive interfaces such as the endothelium [14, 15] and the blood–brain barrier [16, 17]. However, despite promising therapeutic results, the underlying physical mechanisms, particularly those associated with megahertz (MHz) acoustic excitation, have not been fully elucidated [18, 19]. This challenge arises in part from the non-repeatable nature of microbubble-cell interactions and their occurrence within ultrashort time windows, typically on the microsecond scale, which exceeds the frame rates of conventional imaging systems and thus limits the ability to capture the full spatiotemporal dynamics of individual events.

Capturing the rapid, nonlinear oscillations of microbubbles demands imaging modalities capable of resolving ultrahigh-speed dynamics in real time (i.e., during the occurrence of the event). This task simultaneously requires four technical capabilities: sub-microsecond temporal resolution to track oscillation cycles driven by MHz-range ultrasound, micrometer-scale spatial resolution to resolve individual microbubbles, sufficient sequence depth to capture their full temporal evolution over multiple cycles, and single-shot data acquisition of the time-resolved images, given the highly non-repetitive nature of these events, particularly in biologically relevant environments. Among existing approaches, advanced high-resolution microscopy systems typically have modest imaging speed at ~ 100 frames per second (fps) [20–23], insufficient to capture sub-microsecond temporal features. Commercial high-speed cameras offer compact designs and can reach a full-frame imaging speed of up to 10 million fps (Mfps) with sequence depths exceeding 100 frames [24], making them attractive for time-resolved imaging. However, achieving such frame rates requires costly and specialized sensor architectures, such as *in-situ* image storage CCDs, which often exhibit limited optical sensitivity due to a low fill factor [25]. To further increase imaging speeds, techniques such as pixel binning or interleaved exposures are commonly used [26] but further compromise spatial resolution or increase implementation complexity.

To lift the requirement for specialized sensors, ultrahigh-speed framing photography [27–32] offers a solution by distributing time-gated frames to separate spatial regions recorded by one or more two-dimensional (2D) detectors. A landmark development in this field is the Brandaris 128 system [29], which employs a rapidly rotating mirror and an arc of 128 CCD detectors to capture 128 sequential frames at frame rates of up to 25 Mfps. Brandaris 128 has enabled detailed studies of microbubble oscillations [33], collapse dynamics [34], and interactions with biological structures [35]. Although eliminating the need for ultrahigh-speed sensors, Brandaris 128 relies on mechanically complex hardware. Operational challenges, such as heat generation during acquisition that restricts continuous operation to ~ 30 s followed by mandatory cooling periods, further reduce experimental throughput. Finally, the system’s large footprint (150 cm $\times$ 150 cm $\times$ 20 cm) and high cost make it impractical for widespread adoption beyond specialized research laboratories.

To overcome these limitations in optical instrumentation, we present compressed optical-streaking dark-field ultrahigh-speed microscopy (COSDUM), featuring a 0.52-µm spatial resolution, a 6-Mfps imaging speed, and a 144-frame sequence depth, using off-the-shelf cameras and optics. As a compact and economical solution, COSDUM enables real-time visualization of MHz microbubble cavitation dynamics, including oscillation, degasification, and collapse. This imaging modality is further applied to capture the microscale motion of a platelet driven by an adjacent microbubble and the adaptive expansion of a microbubble around a red blood cell (RBC).

## Results

### System and principle of COSDUM

**Fig. 1:**
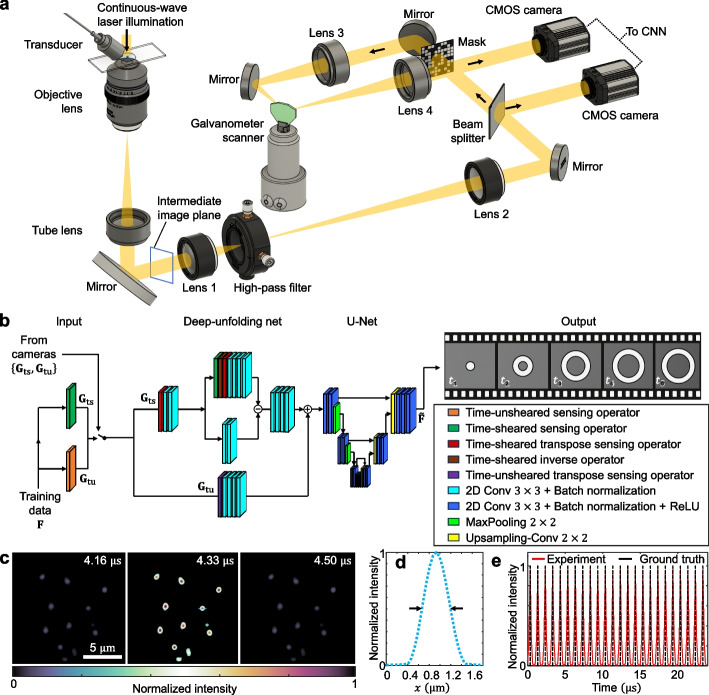
Principle of compressed optical-streaking dark-field ultrahigh-speed microscopy (COSDUM). **a** System schematic. CMOS, complementary metal-oxide semiconductor; CNN, convolutional neural network. **b** CNN schematic. Conv: Convolutional; ReLU: Rectified linear unit. **c** Three representative reconstructed frames showing a 1-MHz laser pulse train incident on 50-nm-diameter nanoparticles. **d** Spatial resolution analysis based on the lateral intensity profile. The distance between the two black arrows corresponds to the full width at half maximum. **e** Temporal intensity profile averaged across the field of view

The COSDUM system is schematically illustrated in Fig. 1a. A 589-nm continuous-wave laser (CNI Lasers, MGL-F-589-200mW) serves as the light source and is directed into an inverted microscope (Olympus, IX73) to provide wide-field illumination to the sample. Encapsulated perfluoropropane microbubbles are insonated by a single-element ultrasound transducer (Olympus, A303S) operating at $f$=1 MHz. The resulting microbubble dynamics are imaged using a 60 $\times$ objective lens (Olympus, LUCPANFL N, NA = 0.7), which transmits the light through a tube lens to the intermediate image plane at the microscope’s output port. The beam is subsequently relayed by a 4*f* imaging system composed of two lenses with focal lengths of 30 mm and 200 mm (Lens 1, Thorlabs, AC254-030-A-ML; Lens 2, Thorlabs, AC254-200-A-ML), providing a 6.67 $\times$ magnification and resulting in a total magnification of 400 $\times$ for the COSDUM system. Dark-field imaging is achieved by placing a 100-µm-diameter high-pass spatial filter (Thorlabs, R1D100P) at the Fourier plane of this 4*f* imaging system to suppress low-frequency components (Supplementary Note S1), thereby enhancing image contrast for microbubble oscillations and increasing spatial sparsity, a condition well suited for compressed sensing. The resulting scene is denoted as $\mathbf{F}\in {\mathbb{R}}^{{N}_{y}\times {N}_{x}\times {N}_{t}}$, where ${N}_{x}\text{ and }{N}_{y}$ represent the data lengths in the two spatial dimensions, and ${N}_{t}$ represents the data length in the temporal dimension.

Then, a beam splitter divides the light in a 90/10 (transmission/reflection) ratio. The reflected light is imaged by a CMOS camera (FLIR, GS3-U3-41C6NIR-C) via spatiotemporal integration (denoted as the operator ${\mathbf{T}}_{\mathrm{tu}}\in {\mathbb{R}}^{{m}_{\mathrm{tu}}\times n}$ with ${m}_{\mathrm{tu}}={N}_{x}{N}_{y}$ and $n={N}_{x}{N}_{y}{N}_{t}$) as the time-unsheared view, whose optical energy is denoted by ${\mathbf{g}}_{\mathrm{tu}}\in {\mathbb{R}}^{{m}_{\mathrm{tu}}\times 1}$. The transmitted component forms an image of the dynamic scene on a transmissive encoding mask (Fineline Imaging, 50% transmission and 22-µm encoding pixel size) with a binary pattern designed using an end-to-end approach (detailed in Supplementary Note S2 and Fig. S1), generating the spatially encoded dynamic scene. This process of spatial encoding is denoted by the operator $\mathbf{C}\in {\mathbb{R}}^{n\times n}$. Then, this image is relayed by a second 4*f* imaging system consisting of two 100-mm focal length lenses (Lens 3 and Lens 4, Thorlabs, AC254-100-A-ML), in which a galvanometer scanner (Thorlabs, GVS201) is positioned at the Fourier plane to introduce temporal shearing, denoted by the operator $\mathbf{S}\in {\mathbb{R}}^{({m}_{\mathrm{ts}}{N}_{t})\times n}$ with ${m}_{\mathrm{ts}}={N}_{y}\left[{N}_{x}+\left({N}_{t}-1\right)\right]$. Finally, the spatially encoded and temporally sheared scene is spatiotemporally integrated (denoted by the operator ${\mathbf{T}}_{\mathrm{ts}}{\in {\mathbb{R}}}^{{m}_{\mathrm{ts}}\times \left({m}_{\mathrm{ts}}{N}_{t}\right)}$) by a second CMOS camera (FLIR, GS3-U3-23S6M-C), producing the time-sheared view, whose optical energy is denoted by ${\mathbf{g}}_{\mathrm{ts}}{\in {\mathbb{R}}}^{{m}_{\mathrm{ts}}\times 1}$.

Overall, COSDUM’s forward model is expressed as

1 $$\begin{array}{c}\mathbf{g}=\left[\begin{array}{c}{\mathbf{g}}_{\mathrm{ts}}\\ {\mathbf{g}}_{\mathrm{tu}}\end{array}\right]=\left[\begin{array}{c}{\mathbf{T}}_{\mathrm{ts}}\mathbf{S}\mathbf{C}\\ {\mathbf{T}}_{\mathrm{tu}}\end{array}\right]\mathbf{f}+\boldsymbol{\epsilon}.\end{array}$$

Here, $\mathbf{f}\in {\mathbb{R}}^{n\times 1}$ is the discrete vectorized representation of the dynamic scene $\mathbf{F}$. ${\mathbf{g}}_{\mathrm{ts}}\in {\mathbb{R}}^{{m}_{\mathrm{ts}}\times 1}$ and ${\mathbf{g}}_{\mathrm{tu}}\in {\mathbb{R}}^{{m}_{\mathrm{tu}}\times 1}$ are the vectorized representations of the time-sheared view ${\mathbf{G}}_{\mathrm{ts}}\in {\mathbb{R}}^{{N}_{y}\times \left[{N}_{x}+\left({N}_{t}-1\right)\right]}$ and the time-unsheared view ${\mathbf{G}}_{\mathrm{tu}}\in {\mathbb{R}}^{{N}_{y}\times {N}_{x}}$, respectively. ${\boldsymbol{\boldsymbol{\epsilon}}}\in {\mathbb{R}}^{m\times 1}$ represents the noise added to the snapshot during data acquisition with $m={m}_{\mathrm{tu}}+{m}_{\mathrm{ts}}$. ${{\boldsymbol{\Phi}}}_{\mathrm{ts}}={\mathbf{T}}_{\mathrm{ts}}\mathbf{S}\mathbf{C}\in {\mathbb{R}}^{{m}_{\mathrm{ts}}\times n}$ is the sensing matrix for the time-sheared view. $\boldsymbol{\Phi} \in {\mathbb{R}}^{m\times n}$ is the COSDUM’s sensing matrix composed of the vertical concatenation of the matrices $\boldsymbol{\Phi }_{\mathrm{ts}}$ and ${\mathbf{\mathrm{T}}}_{\mathrm{tu}}$. Details of COSDUM’s forward model are explained in Supplementary Note S3.

To reconstruct the dynamic scene, the acquired snapshots are processed by a convolutional neural network (CNN)-based algorithm composed of two cascaded modules (Fig. 1b). The first module is a deep unfolding network that incorporates COSDUM’s forward model by mapping a single iteration of the alternating direction method of multipliers (ADMM) [36] onto a structured set of convolutional and sensing operations [37, 38]. Together, these layers approximate the inverse model and generate an initial estimate. The second module is a U-Net [39] with a three-level encoder-decoder structure, which refines the intermediate reconstruction to enhance spatial and temporal fidelity. Leveraging the "splitting-and-optimization" strategy inherent to ADMM, this design achieves high memory efficiency, which is essential for learning to reconstruct the spatiotemporal datacube of the dynamic scene (denoted by $\widetilde{\mathbf{F}}\in {\mathbb{R}}^{{N}_{y}\times {N}_{x}\times {N}_{t}}$). In this regard, COSDUM’s image reconstruction is formulated as

2 $$\begin{array}{c}{\mathrm{minimize}}_{\mathbf{f},\mathbf{z}}{\Vert \mathbf{g}-{\boldsymbol{\Phi}}\mathbf{f}\Vert }_{2}^{2}+\psi \left(\mathbf{z}\right), ~~\text{subject to} ~~~\mathbf{z}=\mathbf{f}.\end{array}$$

Here, $\psi \left(\cdot \right):{\mathbb{R}}^{n\times 1}\to \overline{\mathbb{R} }$ is a regularizer. $\mathbf{z}\in {\mathbb{R}}^{n\times 1}$ is an auxiliary variable.

To learn the CNN’s weights, the loss function $\mathcal{H}\left(\cdot \right):{\mathbb{R}}^{{N}_{y}\times {N}_{x}\times {N}_{t}}\to {\mathbb{R}}^{+}$ is established as

3 $$\begin{array}{c}\mathcal{H}\left(\mathbf{F},\widetilde{\mathbf{F}}\right)={l}_{1}\left(\mathbf{F},\widetilde{\mathbf{F}}\right)+{l}_{\mathrm{SSIM}}\left(\mathbf{F},\widetilde{\mathbf{F}}\right).\end{array}$$

Here, ${l}_{1}(\cdot )$ is the ${l}_{1}$-norm. ${l}_{\mathrm{SSIM}}\left(\cdot \right)=1-\mathrm{SSIM}\left(\mathbf{F},\widetilde{\mathbf{F}}\right)$ represents the structural similarity index measurement (SSIM) loss function [40], where $\mathrm{SSIM}\left(\cdot \right)$ denotes the SSIM metric. The retrieved datacube of the dynamic scene has a sequence depth of ${N}_{t}=144$ frames, each containing up to ${N}_{y}\times {N}_{x}=1024\times 1024$ pixels. A detailed explanation of the image reconstruction algorithm is provided in Supplementary Note S4 and Methods.

The COSDUM system yields a field of view (FOV) of 14.85 µm $\times$ 14.85 µm $,$ calculated based on a camera pixel size of 5.8 µm and the total magnification of 400$\times$. The imaging speed of COSDUM is set to 6 Mfps, determined by the interplay between the galvanometer scanner’s scan rate, the selected focal lengths, and the camera’s pixel size (see details in Methods, Supplementary Note S5, and Fig. S2).

### Quantification of the system’s performance of COSDUM

To experimentally validate the spatial and temporal resolutions of COSDUM, we imaged isolated nanoparticles prepared in-house with a nominal diameter of 50 nm [41], illuminated by a pulsed laser (Spectra-Physics, VPFL-G-HE-30) operating at a repetition rate of 1 MHz with a pulse duration of 3 ns. Due to the sub-diffraction-limit size of these nanoparticles and the nanosecond scale of illumination, the pulsed-laser-illuminated nanoparticles effectively served as a spatiotemporal delta function, making them an ideal probe for characterizing the system’s point spread functions (PSFs) in both spatial and temporal domains. COSDUM recorded 24 consecutive pulses in a single acquisition. The reconstructed video is provided in Movie S1, and three representative frames are presented in Fig. 1c. Figure 1d shows the lateral intensity profile across a selected nanoparticle, from which the spatial resolution, defined as the full width at half maximum (FWHM) of its spatial intensity profile, was measured to be 0.52 µm, in agreement with the theoretical estimate. Figure 1e plots the frame-averaged signal intensity across the FOV. The resulting time trace reveals 24 regularly spaced pulses at 1-µs intervals, confirming COSDUM’s stability and timing accuracy. The average FWHM of each pulse’s temporal intensity profile was 0.33 µs, quantifying the system’s temporal resolution.

### Characterization of microbubble dynamics using COSDUM

We used COSDUM to monitor two distinct cavitation regimes exhibited by insonated microbubbles: stable cavitation and a non-repetitive burst event. The former is characterized by periodic oscillations, whereas the latter involves a sudden, irreversible disruption of the microbubble, resulting in collapse. Details on the synchronization between the ultrasound excitation and COSDUM’s image acquisition and sample preparation are provided in Methods, Supplementary Notes S6, and Figs. S3-S4.

**Fig. 2:**
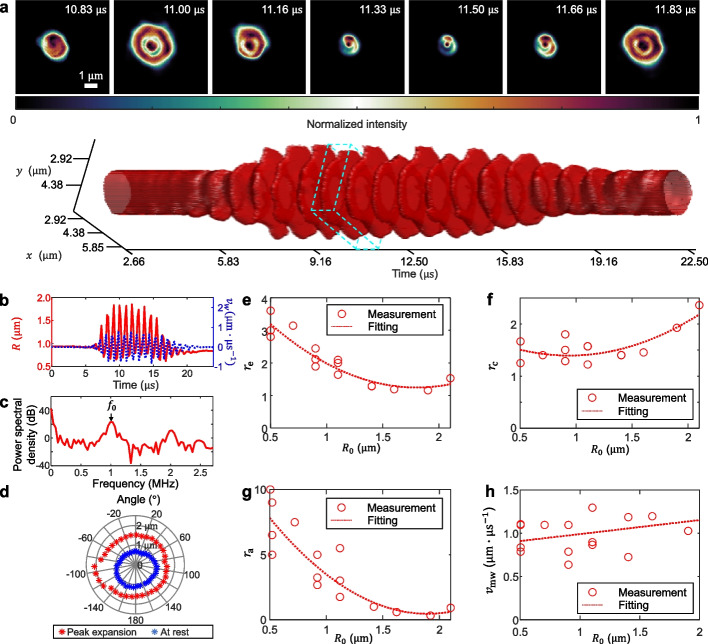
COSDUM of stable cavitation in insonated microbubbles. **a** Time-resolved microbubble radial oscillations with representative frames showing microbubble dynamics in one ultrasound cycle (marked by the cyan dashed cuboid). **b** Quantification of the time-resolved radius (red solid line) and the wall velocity ${v}_{\mathrm{w}}$ (blue dotted line) of the microbubble. **c** Power spectrum of the time-resolved radius extracted from (b). **d** Angular distribution of microbubble radius at rest and at peak expansion over 10 cycles. **e–h** Quantification of the expansion ratio ${r}_{\mathrm{e}}$ (e), the compression ratio ${r}_{\mathrm{c}}$ (f), the oscillation asymmetry ${r}_{\mathrm{a}}$ (g), and maximum wall velocity ${v}_{\mathrm{mw}}$ (h) across 17 measured microbubbles. The red dotted curves in (e)–(g) were obtained by fitting the experimental data with a fourth-order polynomial. The red dotted curve in (h) was obtained by linear regression fitting

As the first demonstration, we insonated a microbubble with the resting radius of ${R}_{0}=$ 0.9 µm using a $f=1$ MHz ultrasound transducer delivering 10 cycles at a pressure of 0.3 MPa. The reconstructed movie and representative frames are shown in Movie S2 and Fig. 2a, highlighting asymmetry between expansion and compression, as well as cycle-to-cycle variations. Figure 2b quantifies the radial oscillation, showing that the microbubble reaches a maximum radius of approximately ${R}_{\mathrm{max}}=$ 1.8 µm and a minimum radius of ${R}_{\mathrm{min}}=$ 0.7 µm, exhibiting preferential expansion over compression. Notably, the microbubble continued to oscillate for two cycles beyond the 10-cycle acoustic pulse, consistent with post-excitation behaviors in a weakly damped medium [42, 43]. A detailed explanation of the microbubble radius estimation procedure is provided in Supplementary Note S7 and Fig. S5.

To further characterize the microbubble dynamics, we calculated the wall velocity ${v}_{\mathrm{w}}=dR/dt$, where $R$ denotes the instantaneous microbubble radius. As shown in Fig. 2b, the results highlight the sharp acceleration during expansion phases. This analysis provides insight into the mechanical stress experienced by the microbubble shell. By the end of the insonification sequence, the microbubble exhibited a $\sim 10\%$ reduction in radius, suggesting net volumetric loss and potential shell disruption or gas diffusion. This interpretation is supported by the frequency-domain analysis, as shown in Fig. 2c, where the power spectra reveal strong energy contributions at both the fundamental (i.e., ${f}_{0}=f$) and second-harmonic (i.e., $2{f}_{0}$) frequencies. Figure 2d compares the angular distribution of the microbubble radius at peak expansion and at rest. The results reveal pronounced anisotropy during expansion, suggesting asymmetric deformation likely caused by uneven acoustic loading.

To quantitatively evaluate COSDUM’s monitoring capabilities, we conducted a statistical analysis of 17 insonated microbubbles with ${R}_{0}$ ranging from 0.5 to 2.1 µm. Figure 2e presents the expansion ratio ${r}_{\mathrm{e}}={R}_{\mathrm{max}}/{R}_{0}$, which decreases with increasing ${R}_{0}$, indicating that smaller microbubbles undergo proportionally greater expansions relative to their resting size. This trend reflects the enhanced deformability and lower inertial resistance of submicrometer-sized microbubbles under ultrasound excitation. Figure 2f shows a slightly increasing trend between ${R}_{0}$ and the compression ratio ${r}_{\mathrm{c}}={R}_{0}/{R}_{\mathrm{min}}$, suggesting that larger microbubbles tend to be more resistant to collapse, possibly due to increased inertial stability or shell stiffness. The asymmetry of the oscillation, quantified by ${r}_{\mathrm{a}}=\left({R}_{\mathrm{max}}-{R}_{0}\right)/ ({R}_{0}-{R}_{\mathrm{min}})$(Fig. 2g), shows a marked decrease with increasing ${R}_{0}$, indicating that smaller microbubbles exhibit more asymmetric oscillations dominated by expansion. This asymmetry is a hallmark of nonlinear acoustic behavior and is associated with enhanced fluid motion and mechanical stress generation in the surrounding medium. Finally, Fig. 2h shows the maximum wall velocity, defined as ${v}_{\mathrm{wm}}=\mathrm{max}\left|{v}_{\mathrm{w}}\right|$, as a function of ${R}_{0}$. No strong dependence is observed, although a slight upward trend is present. This result suggests that oscillation speed is not solely dictated by microbubble size but is likely influenced by a combination of local acoustic conditions, damping effects, and mechanical interactions with the surrounding fluid.

**Fig. 3:**
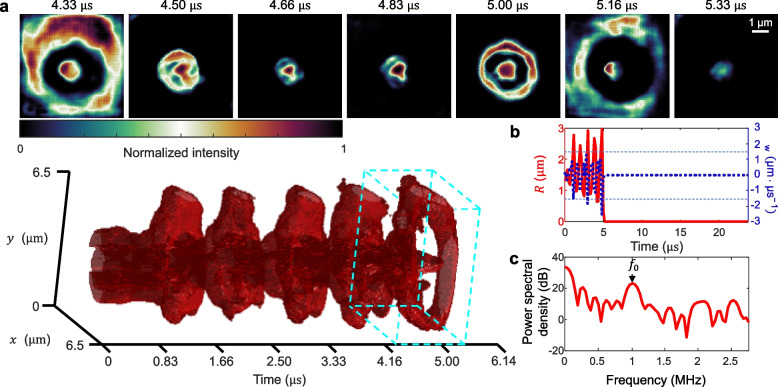
COSDUM of the collapse of an insonated microbubble. **a** Time-resolved microbubble radial oscillations with representative frames showing microbubble dynamics in one ultrasound cycle (marked as the cyan dashed cuboid). **b** Quantification of the radial profile of the microbubble over time (red solid line) with its radial wall velocity profile ${v}_{\mathrm{w}}$ (blue dotted line). **c** Radial power spectrum extracted from (**b**)

To investigate the onset of extreme nonlinear behavior, we increased the acoustic pressure amplitude to 1 MPa to drive a microbubble beyond its stable oscillation regime. The reconstructed evolution is shown in Movie S3 and Fig. 3a. Quantification of the radial oscillation (Fig. 3b) shows that, under elevated excitation, the microbubble underwent rapid expansion followed by collapse during the fifth oscillation cycle. Analysis of its wall velocity ${v}_{\mathrm{w}}$ reveals strong radial oscillations within the first 5 µs, followed by an abrupt drop to zero velocity. The microbubble undergoing inertial collapse exhibits a peak wall velocity significantly above the trend line shown in Fig. 2h, further supporting the idea that excessive wall motion may serve as a predictor of shell failure or gas escape. This comparison underscores the potential of wall velocity as a diagnostic parameter for distinguishing between stable and collapsing microbubbles. Figure 3c shows the power spectrum of the burst event, with a dominant peak at the fundamental frequency (i.e., ${f}_{0}=f$) and a broad, irregular distribution beyond. The lack of harmonic structure and elevated subharmonic content are consistent with inertial cavitation and the loss of periodic oscillation.

### Ultrahigh-speed visualization of microbubble-cell interactions in whole blood

**Fig. 4:**
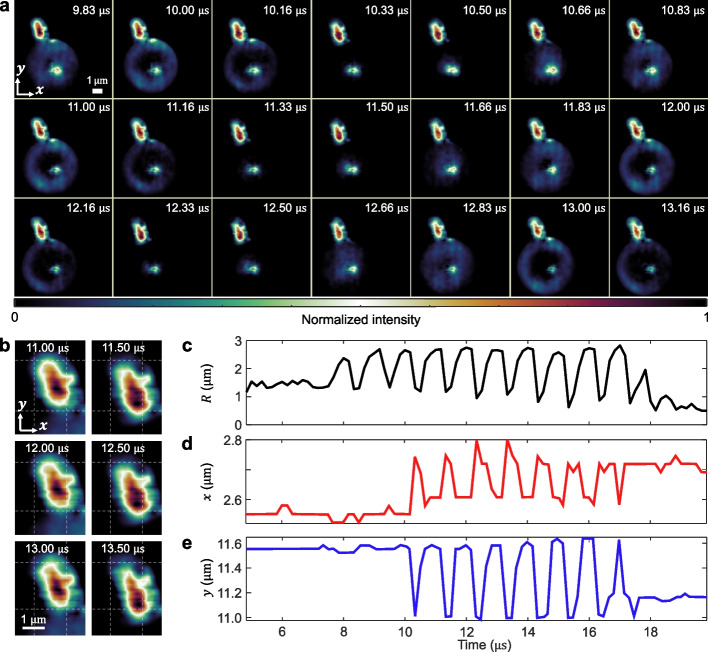
COSDUM of microbubble-platelet interactions in whole blood. **a** Representative reconstructed frames showing the periodic motion of a platelet driven by an uncontacted microbubble. **b** Close-up view of the platelet during three representative oscillation cycles. **c** Quantification of the radial profile of the microbubble over time. **d–e** Central position of the platelet along the $x$- and $y$-axes, respectively

To demonstrate COSDUM’s feasibility in a biologically relevant environment, we investigated the interaction between a stably oscillating microbubble and a nearby platelet in a suspension of microbubbles in whole blood (Charles River Laboratories, PB000F-2-KIT). Microbubbles are actively explored for thrombolysis, with promising pre-clinical and clinical progress [44]. Here, the microbubble was insonated with a 10-cycle, 1-MHz ultrasound pulse at a pressure of 1 MPa. The reconstruction is shown in Movie S4, and selected frames from the ultrahigh-speed sequence (Fig. 4a) illustrate the evolution of the microbubble radius along with the corresponding motion of the platelet over time. To further highlight this interaction, Fig. 4b presents a zoomed-in view of the region surrounding the platelet during three representative oscillation cycles, capturing its position at both maximum expansion and compression of the microbubble. Quantitative analysis of the microbubble radius over time (Fig. 4c) reveals consistent oscillatory behavior without evidence of collapse, confirming sustained stable cavitation throughout the observation window. Concurrently, tracked platelet trajectories along the $x$- and $y$-axes (Fig. 4d and e) exhibit periodic displacements that become synchronized with the microbubble’s oscillations after the third cycle (i.e., ${t}_{\mathrm{th}}=$ 10.33 μs). The maximum displacement reached approximately 0.6 µm, representing a substantial fraction of the platelet’s estimated diameter of 2 µm [45]. This behavior is consistent with oscillation-induced microstreaming [46, 47], which generates steady vortical flows capable of producing localized shear forces sufficient to drive the motion of nearby cellular components. Indeed, platelet accumulation and activation are well-known to be highly dependent on shear-stress mechanosensitive pathways [48, 49]. On the microsecond timescale observed here, we can estimate the local microbubble-induced time-dependent shear stress $\tau (t)$ as

4 $$\begin{array}{c}\tau \left(t\right)=\frac{\mu \omega \left[{R\left(t\right)-R}_{0}\right]}{\delta },\end{array}$$

where $\delta =\sqrt{{}^{2\mu }\!\left/ \!{}_{{\rho }_{\mathrm{fd}}\omega }\right.}$ is the viscous boundary layer [14, 50]. $\mu$, $\omega$, and ${\rho }_{\mathrm{fd}}$ are the dynamic viscosity of the surrounding fluid (0.001 Pa·s), the angular frequency of oscillation ($2\pi f$), and the fluid density (1000 kg/m^3^), respectively. By modeling the platelet motion as a damped harmonic oscillator and assuming a microbubble-platelet contact area on the order of 1 μm^2^ (Fig. 4b), we estimated the platelet mass to be ~ 2.9 $\times$ 10^–15^ kg, consistent with currently accepted indirect values [51] (see details in Supplementary Note S8 and Fig. S6a). This observation provides a first-of-its-kind estimation of the platelet shear activation threshold under MHz insonication, relevant for ultrasound-mediated sonothrombolysis applications. Here, we calculate the shear impulse $I$ given as

5 $$\begin{array}{c}I= \int \tau \left(t\right)dt,\end{array}$$

resulting in a shear impulse threshold of ${I}_{\mathrm{th}}$=3.62 $\times$ 10^–2^ Pa·s (0.362 dyn·s/cm^2^), beyond which platelet motion became influenced by the locally generated microbubble shear flow (see details in Supplementary Note S9 and Fig. S6b).

**Fig. 5:**
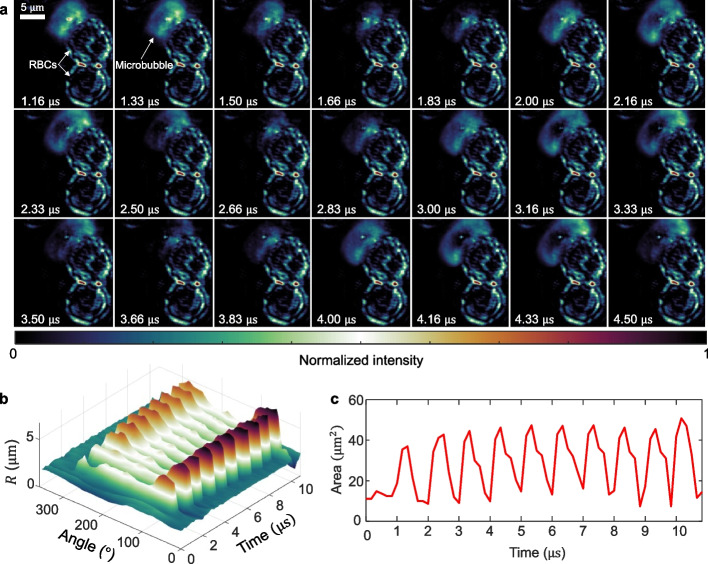
COSDUM of microbubble-red blood cell (RBC) interactions in whole blood. **a** Representative reconstructed frames showing the shape-adapted expansion of a microbubble around an adjacent RBC, resulting in a contact surface of approximately 25% of the RBC. **b** Microbubble radius as a function of angle and time. **c** 2D projected area of the microbubble over time, highlighting periodic oscillations and inter-cycle variations

We next investigated a microbubble positioned adjacent to an RBC during ultrasound excitation. While prior studies have focused on microbubble-vascular interactions, or the interplay between microbubbles and non-hematological cells, direct and dynamic physical interactions of a single microbubble with individual RBCs have not been well documented. Here, the microbubble was insonated with a 10-cycle, 1-MHz pulse at 1 MPa peak negative pressure. The full evolution is shown in Movie S5. Selected frames from the ultrahigh-speed sequence (Fig. 5a) show asymmetric oscillations, suggesting that mechanical coupling with the RBC alters radial dynamics. Angular quantification of the microbubble radius (Fig. 5b) reveals persistent anisotropy across cycles, with directional expansion likely confined by the adjacent RBC. Compared to isolated microbubbles, this constrained behavior may enhance localized shear and fluid motion. The projected area over time (Fig. 5c) exhibits periodic oscillations, consistent with stable cavitation and nonlinear dynamics.

## Discussion

We have developed COSDUM by synergizing compressed sensing, streak imaging, dark-field microscopy, and deep learning to advance computational ultrahigh-speed optical microscopy for capturing nonlinear microbubble dynamics and microscale biophysical interactions in real time. System characterization demonstrated an imaging speed of 6 Mfps, a spatial resolution of 0.52 μm, a temporal resolution of 0.33 μs, and a sequence depth of 144 frames. Applied with a 1-MHz ultrasound transducer, COSDUM monitored encapsulated perfluoropropane microbubbles with resting radii ranging from 0.5 to 2.1 μm. The recorded movies enabled the extraction of key physical parameters, including radial oscillations, wall velocities, and frequency spectra. These results demonstrate that microbubble oscillation dynamics depend strongly on the initial radius. In particular, smaller microbubbles exhibited higher expansion ratios and greater asymmetry in their radial profiles, consistent with increased compliance and lower inertial resistance, which make them more susceptible to acoustic pressure variations [52]. These trends underscore the critical role of microbubble size and mechanical characteristics in shaping their nonlinear acoustic behaviors.

COSDUM offers a new approach to microbubble assessment. Unlike bulky and mechanically complex platforms such as Brandaris 128, COSDUM operates as a compact system (with a footprint of 45 cm $\times$ 30 cm $\times$ 11 cm) with tunable sequence depth and seamless integration into standard microscopy configurations. In contrast to commercial high-speed cameras that require complex auxiliary cooling and are prohibitively expensive for most laboratories, COSDUM offers a fully integrated dual-camera architecture with Mfps-range imaging capability and an image format of > 1 million pixels, all at a significantly lower cost. Compared to the recent advances in ultrahigh-speed imaging using multiplexed light-emitting-diode illumination [53] or acousto-optic frequency sweeping [54], COSDUM exhibits a clear advantage in sequence depth. Its compactness, combined with dark-field imaging, delivers enhanced spatial contrast for weakly scattering microstructures. These technical features of COSDUM have empowered the visualization of key phenomena across a range of microbubble sizes, including stable cavitation, nonlinear radial oscillations, post-excitation free oscillations, and inertial collapse. A comparison between COSDUM and representative modalities that have been, or could be, used to study microbubble dynamics is provided in Supplementary Note S10 and Table S1.

COSDUM’s attractive ability to probe both stable and disruptive microbubble behaviors in a whole-blood environment with high spatiotemporal fidelity offers new mechanistic insights into microscale fluid–structure interactions. COSDUM captured phase-dependent platelet displacements. This observation enables, for the first time, an estimation of the platelet shear activation threshold under MHz insonation. The estimated shear impulse threshold is well below previously reported thresholds for platelet activation and clot formation (e.g*.*, via von Willebrand factor release), which lie in the range of 10–1000 Pa·s [55], albeit acquired under completely different timescales. This unique interaction highlights the fundamental measurements made possible by COSDUM and provides biophysical insight into the modulation of platelet activity under microbubble-mediated ultrasound regimes. COSDUM also captured a microbubble wrapping around an RBC during expansion. This new observation―namely the ability of the microbubble to partially conform to a nearby RBC, increasing the surface area of contact―may have implications for therapeutic delivery (e.g*.*, to RBCs in blood disorders) and modulation of the local immune response through increased spatially integrated forces to promote mechanotransductive signaling (e.g*.*, Piezo1), which in turn can affect RBC physiology [56].

Beyond this demonstration, COSDUM represents a versatile and economical ultrahigh-speed imaging platform with broad applications in real-time biophysical studies, microbubble-mediated ultrasound therapy, and microbubble-assisted drug delivery. Its ability to resolve the spatiotemporal complexity of microbubble-cell interactions will open opportunities for investigating ultrasound-induced clot formation [57], platelet activation [58], thrombolysis [59], and microvascular biomechanics [60]. Moreover, COSDUM’s ability to capture non-repetitive burst events will provide direct access to studying inertial cavitation onset and localized mechanical stresses relevant to therapeutic cavitation [61] and drug delivery strategies [62].

COSDUM’s architecture offers clear opportunities for performance enhancement. Its imaging speed is proportional to the maximum angular scanning speed of the galvanometer scanner. By replacing the current galvanometer scanner with a resonant galvanometer scanner (e.g., Thorlabs, GRS8-AG) [63], the imaging speed could be increased to reach, and potentially exceed, 25 MHz, comparable to those achieved by Brandaris-type systems. This improvement could enable visualization of even faster transient events such as shockwave emission [64] and high-frequency acoustic phenomena in microbubble dynamics [65]. COSDUM’s spatial resolution is mainly determined by the NA of the microscope objective. Using a microscope objective with a larger NA (e.g., Olympus, UPLSAPO 60XW, NA = 1.2) [66] could further improve the spatial resolution, potentially enabling the visualization of subcellular membrane deformation during microbubble oscillation [67]. In this regard, COSDUM’s maximum FOV is determined by the optical field number of the microscope objective in combination with the total system magnification, which together define the maximum accessible imaging area in the sample plane and are ultimately limited by the detector size. A larger sensor (e.g., Teledyne, Adimec S-25A80-Gx) [68] would enable full utilization of the maximum FOV defined by the optical system, while simultaneously influencing the achievable number of frames, which is governed by the interplay between exposure time, frame size, and sensor pixel count. This configuration could enable observation of microbubble dynamics across larger spatial scales and extended temporal windows, such as collective oscillatory behavior [69]. In parallel, upgrading to more sensitive cameras could further improve detection sensitivity. Together, these developments would position COSDUM as a next-generation platform for exploring extreme biomechanical processes, advancing therapeutic ultrasound technologies, and uncovering new biophysical mechanisms at the microscale.

## Materials and methods

### Image reconstruction based on a deep-learning approach

The baseline database was created from the “SumMe” [70], “Need for Speed” [71], and “Sports Videos in the Wild” [72] databases. To train the CNN weights, we randomly selected and cropped 1000 datacubes with ${N}_{x}\times {N}_{y}=1024\times 1024$ pixels and ${N}_{t}=144$ frames. In addition to video-based data, we generated a physics-driven training dataset by simulating microbubble dynamics using the Rayleigh-Plesset equation [73]. This model captures the nonlinear radial oscillations of spherical microbubbles in response to acoustic pressure, providing physically accurate temporal profiles that improve the CNN’s ability to reconstruct realistic dynamics. All simulations were implemented in PyTorch and trained on an Intel Core i9 12900 K 3.2 GHz with a GeForce RTX 3090 GPU (24 GB RAM) using the ADAM optimizer.

### Summary of key system parameters

The galvanometer scanner, positioned at the Fourier plane of the 4*f* imaging system formed by Lens 3 and Lens 4 (Fig. 1a), deflects temporal information to distinct spatial locations. Rotating during data acquisition, it alters the reflection angles of the spatial frequency components corresponding to different time-of-arrival of the frames. Lens 4 then performs a Fourier transform, converting these angular deviations into lateral displacements on the CMOS camera that records the time-sheard view. This spatial shifting encodes temporal information as spatial offsets, thereby achieving temporal shearing. The imaging speed is determined by the data acquisition rate of the time-sheared view. In this work, the reconstructed movie achieves a frame rate of [74, 75].

6 $$\begin{array}{c}r= \frac{\gamma {V}_{\mathrm{g}}{f}_{4}}{{t}_{\mathrm{s}}d}.\end{array}$$

Here, $\gamma =0.093 \mathrm{rad}/\mathrm{V}$ is a constant that relates the galvanometer scanner’s deflection angle to the input voltage waveform with ${V}_{\mathrm{g}}=15 \mathrm{V}$, taking into account the waveform's shape and dynamics. ${f}_{4}=100$ mm is the focal length of Lens 4, ${t}_{\mathrm{s}}$ denotes the period of the sinusoidal voltage waveform applied to the galvanometer scanner, and $d=5.8$ μm is the CMOS sensor’s pixel size. The imaging speed of COSDUM is $r=$ 6 Mfps, a value confirmed by the pulse train transmission experiment shown in Figs. 1c–e and Supplementary Note S5. The sequence depth of COSDUM can be calculated by

7 $$\begin{array}{c}{N}_{t}=r\cdot {t}_{\mathrm{e}}.\end{array}$$

Here, ${t}_{\mathrm{e}}$ is the exposure time of the CMOS camera that records the time-sheared view. For all the experiments presented in this paper, the exposure time was set to ${t}_{\mathrm{e}}=24$ μs, yielding ${N}_{t}=$ 144 frames.

### Details on equipment and synchronization of COSDUM

An arbitrary function generator (Tektronix, AFG 31000) was manually triggered to produce both a sinusoidal signal and a transistor-transistor-logic (TTL) signal. The sinusoidal signal was amplified by a 55-dB power amplifier (Electronics & Innovation, A150 RF) and delivered to the transducer (Olympus, A303S-SU 1 MHz/0.5") for acoustic excitation. The TTL signal was split using a T-connector: one branch triggered the power amplifier, while the other was sent to a delay generator (Stanford Research Systems, DG 645) that output three synchronized TTL pulses. The first two pulses triggered the 24-µs exposures of both CMOS cameras, respectively. The third pulse triggered a second function generator (Rigol, DG 1022Z), configured in external burst mode, which generated a single cycle of a 250-Hz sinusoidal waveform to control the rotation of the galvanometer scanner.

### Details on sample preparation

Microbubble experiments were conducted in a slide-based sample chamber designed to provide optical transparency and stable acoustic coupling. Encapsulated perfluoropropane microbubbles (Lantheus Medical Imaging, Definity™) were first diluted by adding one drop of the commercial microbubble suspension into a 1.5-mL microcentrifuge tube containing distilled water and were then gently mixed to ensure uniform dispersion. A small aliquot of the diluted solution was then deposited onto a standard microscope glass slide. A thin cover glass (VWR, No. 1, 24 × 60 mm) was gently placed over the droplet to confine the microbubbles within a shallow liquid layer and minimize bulk fluid motion during ultrasound excitation.

A customized water tank was positioned over the slide-coverslip assembly and filled with degassed water to provide efficient acoustic coupling. The ultrasound transducer was housed in a water-filled flexible enclosure placed in contact with the tank water and oriented at an angle of 45° relative to the imaging plane to enable oblique insonation while maintaining optical access. The microbubbles were insonicated using a 10-cycle sinusoidal pulse at a center frequency of 1 MHz, generated by the arbitrary function generator and amplified by the 55-dB radio-frequency power amplifier before delivery to the transducer. More details of the ultrasound synchronization are provided in Supplementary Note S6. The peak negative acoustic pressure at the sample plane was calibrated in a separate water tank using a needle hydrophone (Onda Corp., HGL-0200). The acoustic pressure was set to approximately 0.3 MPa for experiments probing stable microbubble oscillations and increased up to 1 MPa for nonlinear excitation and inertial cavitation studies.

## Supplementary Information

Additional file 1.

Additional file 2.

Additional file 3.

Additional file 4.

Additional file 5.

Additional file 6.

## Data Availability

All data supporting the results of this study are available within the paper and its Supplementary Information. Additional data are available for research purposes from the corresponding author upon reasonable request.
