# Supplementary material for: Computational optical streak microscopy of megahertz acoustic microbubble dynamics
Source: Photonix. 2026 May 1;7(1):15. doi: 10.1186/s43074-026-00232-8 (PMC13133243; doi:10.1186/s43074-026-00232-8)
Supplement: Supplementary file 6 — Additional file 6. [file 43074_2026_232_MOESM6_ESM.docx]

Supplementary Information for

**Computational optical streak microscopy of megahertz acoustic microbubble dynamics**

Miguel Marquez, Yingming Lai, Miao Liu, Elahe Memari, Brandon Helfield^*^,

Jinyang Liang^*^

* Corresponding authors: jinyang.liang@inrs.ca (J.L.); brandon.helfield@concordia.ca (B.H.)

**This PDF file includes:**

Supplementary Notes S1 to S10

Figures S1 to S6

Table S1

References S1 to S15

**Other Supplementary Materials for this manuscript include the following:**

Movies S1 to S5

## Supplementary Note S1: Details of high-pass filter design

The high-pass filter in compressed optical-streaking dark-field ultrahigh-speed microscopy (COSDUM) is determined by the spatial-frequency distribution produced when the intermediate image formed by the objective lens and tube lens is Fourier-transformed by Lens 1 (with focal length $f_{1}$) of the first 4*f* relay module (**Fig. 1a**). Let $U(x,y)$ denote the complex optical field at the intermediate image plane (i.e., the front focal plane of Lens 1). The field distribution at the back focal plane of Lens 1 is then given by

$$\begin{aligned} U_{F}\left( \xi,\eta\right)=\frac{1}{i\lambda f_{1}}\iint U\left( x,y \right)\exp\left( -i2\pi\left[ \frac{\xi}{\lambda f_{1}}x+\frac{\eta}{\lambda f_{1}}y \right] \right)dxdy. \#\left( S1 \right) \end{aligned}$$

The spatial frequencies are defined as $f_{x}=\frac{\xi}{\lambda f_{1}}$and $f_{y}=\frac{\eta}{\lambda f_{1}}.$ Thus, a position in the radial direction is linked to the spatial frequencies by

$$\begin{aligned} \rho'=f_{1}\lambda f',\#\left( S2 \right) \end{aligned}$$

where $f'=\sqrt{f_{x}^{2}+f_{y}^{2}}$ and $\rho'=\sqrt{\xi^{2}+\eta^{2}}$.

Spatial filtering in COSDUM is implemented by multiplying the Fourier-plane field $U_{F}\left( \xi,\eta\right)$ by an aperture function $H\left( \xi,\eta\right)$. For dark-field imaging, the implemented binary high-pass filter takes the form of

$$\begin{aligned} H\left( \rho' \right)=\left\{ \begin{matrix} 0, & \rho'<{\rho'}_{cut-on} \\ 1, & \rho'\geq{\rho'}_{cut-on} \end{matrix} , \right. \#\left( S3 \right) \end{aligned}$$

where ${\rho'}_{cut-on}$ is the radius of the opaque central region. The filtered field in the Fourier plane is therefore

$$\begin{aligned} \tilde{U}_{F}\left( \xi,\eta\right)= U_{F}\left( \xi,\eta\right)H\left( \xi,\eta\right).\#\left( S4 \right) \end{aligned}$$

The second lens of the 4$f$ relay, denoted as Lens 2 (with a focal length $f_{2}$), performs another Fourier transform of this filtered distribution. At the back focal plane of Lens 2, the resulting field is

$$\begin{aligned} U_{\mathrm{DF}}\left( x,y \right)=\frac{1}{i\lambda f_{2}}\iint\tilde{U}_{F}\left( \xi,\eta\right)\exp\left( -i2\pi\left[ \frac{x}{\lambda f_{2}}\xi+\frac{y}{\lambda f_{2}}\eta\right] \right)d\xi d\eta. \#\left( S5 \right) \end{aligned}$$

This operation reconstructs a filtered version of the intermediate image.

To determine ${\rho'}_{cut-on}$, we evaluated the spatial-frequency content generated by microbubbles and the extent of the low-frequency illumination components on the Fourier plane. In our experiments, the microbubbles have resting radii between 0.5 $\mu m$ and 2.1 $\mu m,$ and we expected a maximum expansion of up to approximately 5 $\mu m$ under acoustic excitation, which produces object-space spatial frequencies on the order of $f_{\mathrm{obj}}\sim1/(2R)$, where $R$ is the instantaneous microbubble radius. After magnification by the 60$\times$objective lens, these frequencies appear in the intermediate image plane as $f_{\mathrm{img}}=f_{\mathrm{obj}}/60$. Based on Eq. S2, the microbubble spatial frequencies are distributed over the Fourier-plane diameter

$$\begin{aligned} {d'}_{\mathrm{bubble}}= 2f_{1}\lambda f_{\mathrm{img}}\approx58-590 \mu m .\#\left( S6 \right) \end{aligned}$$

Based on Eq. S6, a set of off-the-shelf high-pass spatial filters, with diameters ranging from 50 to 400 μm, was evaluated. The one with the 100-µm diameter (Thorlabs, R1D100P) provided the best balance between optical throughput and image sharpness and was therefore selected for COSDUM experiments.

## Supplementary Note S2: Comparison between the designed mask and a pseudo-random binary mask

The encoding mask employed in the COSDUM system is designed based on a differentiable learning strategy inspired by Ref. [S1]. In particular, a customized layer is placed at the beginning of the neural network and functions as a learnable coded aperture. This layer generates a two-dimensional (2D) array of trainable parameters $\theta_{i_{y},i_{x}}$, where $i_{x}=\left\{ 0,\ldots,N_{x}-1 \right\}$ and $i_{y}=\left\{ 0,\ldots,N_{y}-1 \right\}$. Each $\theta_{i_{x},i_{y}}$ is initialized with a random value drawn from a uniform distribution and is optimized through backpropagation along with the network weights. To enable gradient-based learning while ensuring output values between 0 and 1, each parameter is passed through a sigmoid function $R_{i_{y},i_{x}}=\left( 1+e^{-\theta_{i_{y},i_{x}}} \right)^{-1},$ where $R_{{i_{y},i}_{x}}$ represents the value at position $\left( i_{y},i_{x} \right)$ on the mask $\mathbf{R}\in\mathbb{R}^{N_{y}\times N_{x}}$. This mask is then propagated through the time-sheared operators, implemented as the green, red, and brown layers in **Fig. 1b**. To promote binarization during training, a regularization term $\sum_{i_{y},i_{x}} \left( R_{i_{y},i_{x}} \right)^{2}\left( R_{i_{y},i_{x}}-1 \right)^{2}$ is added to the overall loss function. This term reaches minima only when $R_{i_{y},i_{x}}=1$ or 0, thus encouraging convergence toward binary values. After training, a threshold of 0.5 is applied to produce the final binary encoding mask.

This approach enables the encoding mask to be jointly optimized with the reconstruction network in a fully differentiable, end-to-end manner. Once optimized, it can be reused in subsequent models without additional retraining. Toward this end, the customized learnable mask layer is removed from the network. The final binary mask, treated as a fixed input, is then directly integrated into the time-sheared sensing operators, ensuring consistent and reproducible sensing in future training or inference pipelines.

The Fourier spectra of a pseudo-random binary mask (with 50% transmittance) and the designed mask are shown in **Fig. S1a**. Compared with the results of the pseudo-random binary mask, the designed mask shows less content in the low-frequency range in its Fourier spectrum and a lower average cross-correlation coefficient. To better comprehend the influence of the mask design on the information distribution on the sensor, we calculated the average pixel intensity in the snapshot (without high-pass filtering) for both a pseudo-random mask and the designed mask (**Fig. S1b**). For this analysis, we used a datacube of all-ones as the target with dimensions of $N_{y}\times N_{x}\times N_{t}=$1024$\times$1024$\times$144 pixels. For the pseudo-random mask encoding, we calculated the average pixel intensity over 1000 executions (each with a different pseudo-random binary mask with 50% transmittance). These results show a more uniform pixel intensity distribution and a higher dynamic range with the designed mask, which echoes the claims presented in Ref. [S1]. Thus, the designed mask results in a more incoherent encoding operation, contributing to improved image quality in reconstruction.

## Supplementary Note S3: Details of COSDUM’s forward model

In the forward model of COSDUM, the spatial encoding matrix $\mathbf{C}\in\mathbb{R}^{n\times n}$, the temporal shearing matrix $\mathbf{S}\in\mathbb{R}^{\left( m_{\mathrm{ts}}N_{t} \right)\times n}$, and the spatiotemporal integration matrices $\mathbf{T}_{\mathrm{ts}}\in\mathbb{R}^{m_{\mathrm{ts}}\times\left( m_{\mathrm{ts}}N_{t} \right)}$ and $\mathbf{T}_{\mathrm{tu}}\in\mathbb{R}^{m_{\mathrm{tu}}\times n}$ are defined as

$$\begin{aligned} C_{j^{'},j}=\left\{ \begin{matrix} \begin{matrix} r_{v} & \mathrm{if}j^{'}=j \end{matrix} \\ \begin{matrix} 0 & \mathrm{otherwise} \end{matrix} \end{matrix} \right. , \#\left( S7 \right) \end{aligned}$$

$$\begin{aligned} S_{i,j}=\left\{ \begin{matrix} \begin{matrix} 1 & \mathrm{if}i=j+N_{y}\cdot N_{t}\cdot\left\lfloor\frac{j}{N_{x}\cdot N_{y}} \right\rfloor\end{matrix} \\ \begin{matrix} 0 & \mathrm{otherwise} \end{matrix} \end{matrix} \right., \#\left( S8 \right) \end{aligned}$$

$$\begin{aligned} \mathbf{T}_{\mathrm{ts}}=\mathbf{1}_{N_{t}}^{T}\otimes\mathbf{I}_{m_{\mathrm{ts}}\times m_{\mathrm{ts}}},\#\left( S9 \right) \end{aligned}$$

and

$$\begin{aligned} \mathbf{T}_{\mathrm{tu}}=\mathbf{1}_{N_{t}}^{T}\otimes\mathbf{I}_{m_{\mathrm{tu}}\times m_{\mathrm{tu}}}.\#\left( S10 \right) \end{aligned}$$

Here, $v=mod(j,N_{x}N_{y})$, $i=\left\{ 0,\ldots,N_{t}m_{\mathrm{ts}}-1 \right\}$, $\left\{ j,j^{'} \right\}=\left\{ 0,\ldots,n-1 \right\}$, $n=N_{x}N_{y}N_{t}$, $m_{\mathrm{ts}}=N_{y}\left[ N_{x}+\left( N_{t}-1 \right) \right]$ and $m_{\mathrm{tu}}=N_{x}N_{y}$. $\otimes$ denotes the Kronecker product. $N_{x}$and$N_{y}$ denote the data lengths in the two spatial dimensions, and $N_{t}$ represents the data length in the temporal dimension. In Eq. S7, $r_{v}$ is the value at the $v$^th^ position of $\mathbf{r}\in\mathbb{R}^{\left( N_{x}N_{y} \right)\times1}$, which is the vectorized form of the designed encoding mask $\mathbf{R}\in\mathbb{R}^{N_{y}\times N_{x}}$ in which $r_{v}\in\left\{ 0, 1 \right\}$. In Eq. S8, $\mathbf{S}$ is a block diagonal matrix. In Eq. S9 and S10, $\mathbf{1}_{N_{t}}\in\mathbb{R}^{N_{t}\times1}$is an all-ones vector. $\mathbf{I}_{m_{\mathrm{ts}}\times m_{\mathrm{ts}}}\in\mathbb{R}^{m_{\mathrm{ts}}\times m_{\mathrm{ts}}}$ and $\mathbf{I}_{m_{\mathrm{tu}}\times m_{\mathrm{tu}}}\in\mathbb{R}^{m_{\mathrm{tu}}\times m_{\mathrm{tu}}}$ are identity matrices.

## Supplementary Note S4. Details of COSDUM’s image reconstruction algorithm

To solve the constrained optimization problem in Eq. 2 in Main Text, we define the augmented Lagrangian (AL) [S2] function as

$$\begin{aligned} \mathcal{L}\left( \mathbf{f,z,w} \right)=\left\| \boldsymbol{g-\Phi f} \right\|_{2}^{2}+\frac{\rho}{2}\left\| \mathbf{f-z+w} \right\|_{2}^{2}+\psi\left( \mathbf{z} \right),\#\left( S11 \right) \end{aligned}$$

where $\rho\geq0$ is the AL penalty parameter. $\mathbf{g}\in\mathbb{R}^{m\times1}$ and $\boldsymbol{\Phi\in}\mathbb{R}^{m\times n}$ are COSDUM’s measurement and sensing matrix in a concatenated form with $m=m_{\mathrm{tu}}+m_{\mathrm{ts}}$ (see Eq. 1 in Main Text for details). $\mathbf{f}\in\mathbb{R}^{n\times1}$ is the discrete vector representation of the dynamic scene. $\mathbf{w}\in\mathbb{R}^{n\times1}$ is the Lagrange multiplier vector. $\psi\left( \cdot\right):\mathbb{R}^{n\times1}\to\bar{\mathbb{R}}$ is a regularizer. $\mathbf{z}\in\mathbb{R}^{n\times1}$ is an auxiliary variable that serves as the argument of $\psi\left( \cdot\right)$. Following the alternating direction method of multipliers (ADMM) [S3] approach, Eq. S11 is decoupled into two analytical inverse models as

$$\tilde{\mathbf{f}}=\mathrm{argmin}_{\mathbf{f}}\left\| \mathbf{g}-\boldsymbol{\Phi f} \right\|_{2}^{2}+\frac{\rho}{2}\left\| \mathbf{f}-\mathbf{z}+\mathbf{w} \right\|_{2}^{2}, \left( S12 \right)$$

and

$$\begin{aligned} \tilde{\mathbf{z}}=\mathrm{argmin}_{\mathbf{z}} \psi\left( \mathbf{z} \right)+\frac{\rho}{2}\left\| \mathbf{f}-\mathbf{z}+\mathbf{w} \right\|_{2}^{2}. \#\left( S13 \right) \end{aligned}$$

To integrate this ADMM structure into the convolutional neural network (CNN) for COSDUM’s image reconstruction, $\mathbf{f}$ is updated by solving the quadratic subproblem in Eq. S12, and $\mathbf{z}$ is approximated using a U-Net [S4]. In Eq. S12, the analytical inverse model of $\mathbf{f}$ refers to a quadratic problem with the closed-form solution $\left[ \boldsymbol{\Phi}^{T}\boldsymbol{\Phi+}\frac{\rho}{2}\mathbf{I} \right]^{-1}\left[ \boldsymbol{\Phi}^{T}\mathbf{g}+\frac{\rho}{2}\left( \mathbf{z}-\mathbf{w} \right) \right]$, where $\boldsymbol{I\in}\mathbb{R}^{m\times m}$ is an identity matrix. In addition, Eq. S13 is a denoising problem, which can be solved by using various denoisers, e.g., block-matching [S5] and 3D filtering (BM3D) [S6]. To solve the first inverse model in Eq. S12 and simplify the process to a smaller-scale matrix inversion, the Sherman Woodbury Morrison (SWM) [S7] matrix inversion lemma, and the full-column rank properties are exploited to obtain the closed-form solution

$$\begin{aligned} \mathbf{f}=\tilde{\rho}^{-1}\left[ \mathbf{I}-\boldsymbol{\Phi}^{T}{[\tilde{\rho}\mathbf{I}+\boldsymbol{\Phi}\boldsymbol{\Phi}^{T}]}^{-1}\boldsymbol{\Phi} \right] \left[ \boldsymbol{\Phi}^{T}\mathbf{g}+\tilde{\rho}\left( \mathbf{z}-\mathbf{w} \right) \right], \#\left( S14 \right) \end{aligned}$$

where $\boldsymbol{\Phi}\boldsymbol{\Phi}^{T}\in\mathbb{R}^{m\times m}$ is a matrix product resulting in a sparse matrix, and $\tilde{\rho}=\rho/2$.

To construct the CNN-based algorithm using Eq. S14, the first step is to define the operators of COSDUM’s data acquisition using the spatiotemporal scene, the binary mask, and temporal shearing. The direct sensing operators of the time-sheared view and the time-unsheared view, denoted by $\mathcal{G}_{\mathrm{ts}}$ and $\mathcal{G}_{\mathrm{tu}}$, are expressed as

$$\begin{aligned} \mathcal{G}_{\mathrm{ts}}\left( \mathbf{F},\mathbf{R} \right)=\sum_{l=0}^{N_{t}-1} \mathcal{R}\left( l, \mathbf{F̅}_{:,:,l}\circ\mathbf{R} \right),\#\left( S15 \right) \end{aligned}$$

and$\begin{aligned} \mathcal{G}_{\mathrm{tu}}\left( \mathbf{F} \right)=\sum_{l=0}^{N_{t}-1} \mathbf{F̅}_{:,:,l},\#\left( S16 \right) \end{aligned}$

Here, $\mathcal{G}_{\mathrm{ts}}\left( \cdot\right):\mathbb{R}^{N_{y}\times N_{x}\times N_{t}}\to\mathbb{R}^{N_{y}\times[N_{x}+\left( N_{t}-1 \right)]}$ and $\mathcal{G}_{\mathrm{tu}}\left( \cdot\right):\mathbb{R}^{N_{y}\times N_{x}\times N_{t}}\to\mathbb{R}^{N_{y}\times N_{x}}$. They are respectively shown as the green and orange layers in **Fig. 1b**. $\mathbf{F}\in\mathbb{R}^{N_{y}\times N_{x}\times N_{t}}$ is the matrix representation of $\mathbf{f}$. ∘ denotes the Hadamard product. The operator $\mathcal{R}\left( \cdot\right):\mathbb{R}^{N_{y}\times N_{x}}\to\mathbb{R}^{N_{y}\times[N_{x}+\left( N_{t}-1 \right)]}$ introduces right-zero-padding (i.e., ${[\mathbf{F}}_{:,:,l},\mathbf{0}]$ with $\mathbf{0}\in\mathbb{R}^{N_{y}\times\left( N_{t}-1 \right)}$) followed by a right-horizontal circular shift of $l$ pixels.

Then, the transpose sensing operators of the time-sheared view and the time-unsheared view, respectively shown as the red and purple layers in **Fig. 1b**, are defined as

$$\begin{aligned} \mathcal{T}_{\mathrm{ts}}\left( \mathbf{G}_{\mathrm{ts}}\mathbf{,R} \right)_{:,:,l}\mathcal{=L}\left( l,\mathbf{G}_{\mathrm{ts}}\mathcal{\circ R}\left( l,\mathbf{R} \right) \right) ,\#\left( S17 \right) \end{aligned}$$

and

$$\begin{aligned} \mathcal{T}_{\mathrm{tu}}\left( \mathbf{G}_{\mathrm{tu}} \right)_{\mathbf{:,:,}l}=\mathbf{G}_{\mathrm{tu}} .\#\left( S18 \right) \end{aligned}$$

Here, $\mathcal{T}_{\mathrm{ts}}\left( \cdot\right):\mathbb{R}^{N_{y}\times[N_{x}+\left( N_{t}-1 \right)]}\to\mathbb{R}^{N_{y}\times N_{x}}$ and $\mathcal{T}_{\mathrm{tu}}\left( \cdot\right):\mathbb{R}^{N_{y}\times N_{x}}\to\mathbb{R}^{N_{y}\times N_{x}}$. They return a datacube from a 2D compressed measurement. $\mathbf{G}_{\mathrm{ts}}\in\mathbb{R}^{N_{y}\times\left( N_{x}+N_{t}-1 \right)}$ and $\mathbf{G}_{\mathrm{tu}}\in\mathbb{R}^{N_{y}\times N_{x}}$ are the matrix representations of the time-sheared view and time-unsheared view measurement (see Eq. 1 in Main Text), respectively. $\mathcal{L}\left( \cdot\right)$ is an operator that performs a left-horizontal circular shift of $l$ pixels, followed by the removal of the last $(N_{t}-1)$ columns in the resulting shifted matrix to preserve the spatial dimension of the datacube.

Finally, the inverse operator of the time-sheared view, shown as the brown layer in **Fig. 1b**, is defined as

$$\begin{aligned} \mathcal{I}_{\mathrm{ts}}\left( \mathbf{G}_{\mathrm{ts}}\mathbf{,R} \right)=\mathbf{G}_{\mathrm{ts}}\circ\left( \sum_{l=0}^{N_{t}-1} \mathcal{R}\left( l,\mathbf{R}^{\circ2} \right)+\tilde{\rho}\mathbf{I} \right)^{\circ-1}.\#\left( S19 \right) \end{aligned}$$

Here, $\mathcal{I}_{\mathrm{ts}}\left( \cdot\right):\mathbb{R}^{N_{y}\times\left[ N_{x}+\left( N_{t}-1 \right) \right]}\to\mathbb{R}^{N_{y}\times[N_{x}+\left( N_{t}-1 \right)]}$. ${(\cdot)}^{\circ2}$ and ${(\cdot)}^{\circ-1}$ represent the Hadamard quadratic power and the Hadamard inverse operation, respectively.

To solve the time-sheared view using the SWM matrix approach (Eq. S14), the model is now reformulated by splitting it into two main equations

$$\begin{aligned} {\boldsymbol{\Phi}_{\mathrm{ts}}}^{T}\mathbf{g}_{\mathrm{ts}}+\tilde{\rho}\left( \mathbf{z}-\mathbf{w} \right),\#\left( S20 \right) \end{aligned}$$

and

$$\begin{aligned} \tilde{\rho}^{-1}\mathbf{I}-\tilde{\rho}^{-1}{\boldsymbol{\Phi}_{\mathrm{ts}}}^{T}\left[ \tilde{\rho}\mathbf{I}+\boldsymbol{\Phi}_{\mathrm{ts}}{\boldsymbol{\Phi}_{\mathrm{ts}}}^{T} \right]^{-1}\boldsymbol{\Phi}_{\mathrm{ts}}.\#\left( S21 \right) \end{aligned}$$

Here, $\boldsymbol{\Phi}_{\mathrm{ts}}\boldsymbol{=}\mathbf{T}_{\mathrm{ts}}\mathbf{SC}$ (see Eq. 1 in Main Text). To reflect the COSDUM sensing model within the neural network architecture, we implement a two-stage design that mirrors the physics-driven formulation. The first stage models the initialization process (Eq. S20) using a deep unfolding approach. It begins with a transpose sensing operator followed by two 2D convolutional layers, each equipped with batch normalization (denoted by Conv+BN and highlighted in cyan in **Fig. 1b**). The result is given as input to the inverse step (Eq. S21), which is divided into two parallel computational paths. The upper path corresponds to the term ${\tilde{\rho}^{-1}\boldsymbol{\Phi}_{\mathrm{ts}}}^{T}{[\tilde{\rho}\mathbf{I}+\boldsymbol{\Phi}_{\mathrm{ts}}{\boldsymbol{\Phi}_{\mathrm{ts}}}^{T}]}^{-1}\boldsymbol{\Phi}_{\mathrm{ts}}$ and consists of a time-sheared sensing operator, followed by the time-sheared inverse operator, then a time-sheared transpose sensing operator and four additional Conv+BN layers. The lower path models the term $\tilde{\rho}^{-1}\mathbf{I}$ using a sequence of two Conv+BN layers. The outputs from both paths are subtracted and passed through four additional Conv+BN layers to generate the initial estimate of the time-sheared path. Next, a third path composed of the time-unsheared transpose sensing operator, followed by four Conv+BN layers, produces supplemental information that is added to the time-unsheared path to obtain the initial estimation of the datacube.

This initialization is passed to a U-Net designed to refine the reconstruction, corresponding to Eq. S13. The U-Net comprises a three-level encoder-decoder structure with skip connections linking the final Conv+ReLU+BN layer in each encoder block (shown in blue in **Fig. 1b**) to the upsampling layers of the decoder (shown in yellow in **Fig. 1b**). The network output is produced via a final 2D convolutional layer with sigmoid activation, yielding the reconstructed datacube.

## Supplementary Note S5: Details of COSDUM’s imaging speed characterization

To experimentally validate the frame rate of COSDUM, we illuminated a narrow slit (Thorlabs, VA100) with a pulsed laser (1 MHz repetition rate; 3 ns pulse width) and recorded the resulting signal using an exposure time of $t_{e}$=24 μs. As shown in **Fig. S2a,** the system captured 24 discrete light pulses, each separated by 1 μs, corresponding to the laser’s repetition rate. The temporal shearing of the signal across the detector is evident as regularly spaced vertical streaks. In **Fig. S2b**, the signal intensity was averaged across rows to produce a 1D temporal profile, revealing 24 well-separated peaks. **Figure S2c** shows the pixel positions of these peaks as a function of pulse index. A linear fit to the data demonstrates uniform temporal spacing between frames, thereby confirming a constant shearing rate. From the slope of this fit, the system’s frame rate was calculated to be 6 Mfps.

## Supplementary Note S6: Ultrasound synchronization

To validate the synchronization between the ultrasound excitation and COSDUM's optical acquisition, we conducted a delay-scanning experiment using a single-cycle, 1 MHz ultrasound pulse with an amplitude of 0.3 MPa. A digital delay generator (Stanford Research Systems, DG645) was used to precisely control the trigger delay between the ultrasound pulse and COSDUM’s image acquisition. Temporally sheared images of a perfluoropropane microbubble were captured at incrementally adjusted delays. As shown in **Fig. S3a**, each COSDUM snapshot reveals a localized distortion along the shearing axis, marking the arrival time of the acoustic excitation within the 24-µs window. **Figure S3b** presents a zoomed-in view of selected frames, illustrating how the ultrasound wavefront reaches the microbubble at different axial positions, as indicated by the red dashed lines. These lines mark the wavefront position at various delay settings. This observed distortion confirms the synchronization accuracy and sub-microsecond delay control. **Figure S3c** quantifies the ultrasound-microbubble interaction by plotting the column index corresponding to the insonified position of the microbubble as a function of delay time. The observed linear relationship confirms that the insonified region shifts at a constant spatiotemporal rate, consistent with COSDUM’s imaging speed (i.e., 6 Mfps).

To further demonstrate COSDUM’s temporal coverage across the full acoustic excitation window, we conducted a second experiment using a 10-cycle, 1 MHz ultrasound pulse. A stationary slit was used to isolate part of a microbubble for dynamic analysis. Two datasets were acquired, one with the ultrasound transducer “off” and one with it “on”, both using a 24-µs exposure to capture the pre-insonation, insonation, and post-insonation phases. The time-unsheared view provides full-field spatial context, while the time-sheared view records the temporal evolution of the isolated part of the microbubble. **Figures S4a** and **b** show that ultrasound exposure induces visible temporal modulations, which are absent in the ultrasound transducer-off condition. Analysis of horizontal line profiles from the time-sheared views (**Fig. S4c**) and the linearly fitted positions of pulse peaks (**Fig. S4d**) confirm consistent timing across 10 oscillation cycles.

## Supplementary Note S7: Comparison of microbubble imaging in bright-field and dark-field imaging modes

To validate the calculation of microbubble size in dark-field imaging mode, we imaged a representative microbubble using both dark-field and bright-field imaging modes by keeping or removing the high-pass filter in the COSDUM system. The results are shown in **Figs. S5a** and **b**. The microbubble radius was estimated by first identifying the centroid and then measuring the radial distance from the centroid to the edge. The comparison is shown in **Figs. S5c** and **d**. The mean radii of these contours are 1.67 ± 0.07 µm (mean ± standard deviation) in bright-field imaging mode and 1.61 ± 0.06 µm in dark-field imaging mode. The mean percentage error between the two profiles was 3.7 ± 2.0 %. The low error indicates strong agreement between the two imaging modes for radius estimation.

## Supplementary Note S8: Modeling of platelet dynamics

Platelet activity is known to be shear-stress-threshold dependent, and our observations (**Fig. 4**) depict approximately 3 cycles of microbubble-induced shear stress before the commencement of platelet motion. To estimate this threshold, cumulative platelet displacement was plotted against time (**Fig. S6a**). The threshold time $t_{\mathrm{th}}$, at which platelet motion began, was estimated as the time at which the derivative of the cumulative displacement rose significantly (~10 times the standard deviation) above the average baseline (~0.08 ± 0.07 µm/µs). The shear impulse $I$, calculated from Eqs. 4 and 5 in Main Text and evaluated at this time point, was considered as the shear impulse threshold $I_{\mathrm{th}}$.

To place the experimental behavior in context, we modeled the oscillatory platelet motion via a forced second-order damped equation of motion, namely,

$$\begin{aligned} m_{p}\ddot{x}(t)+c\dot{x}(t)+k_{s}x(t)=F\left( t \right),\#\left( S22 \right) \end{aligned}$$

where $m_{p}$, $c$, and $k_{s}$ denote the platelet mass, damping constant, and spring constant, respectively. The external driving force, $F(t)$, was estimated from the neighboring microbubble dynamics and contact area (detailed in the section on *Ultrahigh-speed visualization of microbubble-cell interactions in whole blood* in Main Text). Fitting Eq. S22 using MATLAB from $t\geq11$ µs (post-threshold) yielded a platelet mass $m_{p}=$2.9$\times$10^-15^ kg, $c=$1.8$\times$10^-9^ kg/s, and $k_{s}=3.1\times$10^-2^ N/m, with an $R$-squared value of 0.82 and a root mean squared error (RMSE) of 3.5$\times$10^-9^ (**Fig. S6b**).

## Supplementary Note S9: Modeling of microbubble dynamics using the Rayleigh-Plesset equation

To generate synthetic training data for the deep learning framework, we numerically solved the Rayleigh-Plesset (RP) equation [S8], which models the radial dynamics of gas-filled microbubbles under ultrasound excitation. The modified RP formula incorporates contributions from liquid inertia, viscosity, surface tension, and external acoustic pressure:

$$\begin{aligned} \rho_{w}R\ddot{R}+\frac{3}{2}\rho_{w}\dot{R}^{2}=p_{\mathrm{gn}}\left( \frac{R_{0}}{R} \right)^{\kappa}+p_{\nu}-P_{0}-\frac{2\sigma}{R}-\frac{4\mu}{R}\dot{R}-p\left( t \right).\#\left( S23 \right) \end{aligned}$$

Here, $R$ denotes the instantaneous microbubble radius and is a function of time $t$. $R_{0}$ is the equilibrium radius of the microbubble. $\rho_{w}=1000 kg/m^{3}$, $\mu=0.001 Pa\cdot s$, and $\sigma=0.015 N/m$ are the density, viscosity, and surface tension of water, respectively. The polytropic exponent was set to $\kappa=1.07$. The ambient and vapor pressures were fixed at $P_{0}=101,325 Pa$ and $p_{\nu}=2,300 Pa$. The internal gas nucleus pressure at rest is expressed as

$$\begin{aligned} p_{\mathrm{gn}}=\frac{2\sigma}{R_{0}}+P_{0}-p_{\nu}.\#\left( S24 \right) \end{aligned}$$

The acoustic pressure waveform was modeled as

$$\begin{aligned} p\left( t \right)=-p_{a}\sin\left( 2\pi ft \right),\#\left( S25 \right) \end{aligned}$$

where the ultrasound frequency was set to $f=1 MHz$, and the peak pressure amplitude $p_{a}$ ranged from 0.3 MPa to 1 MPa. To reflect experimentally relevant size distributions, we varied $R_{0}$ over a range of 0.5–2.1 µm. Each simulation spanned a 24-µs time window to match COSDUM’s optical acquisition. The resulting radius-time profiles captured a variety of nonlinear behaviors, including stable oscillations and collapse, forming the basis for a diverse synthetic training dataset.

## Supplementary Note S10: Comparison of COSDUM with existing single-shot imaging modalities operating in the million-frame-per-second (Mfps) regime

To articulate the differences between COSDUM and existing single-shot imaging modalities with comparable speed that have been, or could be, used to study microbubble dynamics, we summarize their technical specifications and representative characteristics in **Table S1**. To explain the details included in this table, we first outline the working principles of these imaging schemes and then discuss their performance in terms of imaging speed, sequence depth, spatial resolution, and practical implementation.

*Framing ultrahigh-speed photography***:** Framing ultrahigh-speed photography systems achieve high imaging speeds by optically distributing a rapid sequence of images onto multiple sensors or separate segments on a single sensor in either the spatial domain or the spatial-frequency domain [S9]. Three representative systems in this category are Brandaris 128 [S10], diffraction-gated real-time ultrahigh-speed mapping (DRUM) photography [S11, S12], and frequency recognition algorithm for multiple exposures (FRAME) [S13]. Brandaris 128, one of the benchmark systems for microbubble imaging, is capable of capturing transient phenomena at up to 25 Mfps. It uses an array of synchronized intensified CCD cameras to record sequentially with a precision of up to 40 ns. The system’s unique optical path layout and precision delay circuitry allow for the acquisition of up to 128 consecutive frames in a single experimental run. Although Brandaris 128 ensures high imaging fidelity and offers one of the highest imaging speeds available, it involves a more complex and costly setup.

DRUM photography [S11, S12] is inspired by the space-time duality that operates by introducing time-space coupling into the diffraction of light. This coupling is realized through a time-varying linear phase ramp applied in the spatial-frequency domain. In practice, the dynamic phase modulation is implemented via the inter-pattern transitions of a digital micromirror device (DMD), which functions as a programmable diffraction grating. DRUM photography can capture transient events in a single exposure at 9.8 Mfps with a temporal resolution of 0.37 µs, and a sequence depth of 13 frames. DRUM photography provides a flexible platform for ultrahigh-speed imaging built from readily available components. The use of a mass-produced DMD to generate the diffraction gate without mechanical movement results in a cost-effective and mechanically stable system.

FRAME with fast light-emitting-diode (LED) illumination [S13] accomplishes single-exposure acquisition at up to 4.56 Mfps. In this approach, spatially encoded white-light LED pulses are combined into a collinear pulse train, where each pulse is synchronized with delays as short as 220 ns. When the spatially modulated images are recorded on the sensor, the Fourier transform of the captured image exhibits multiple distinct clusters corresponding to the different illumination pulses, resulting in the acquisition of up to four consecutive frames. Although the setup is relatively simple and cost-effective, the sequence depth is limited by the number of LEDs and the precision of the synchronization electronics. To extend the achievable sequence depth, a DMD was later incorporated to generate a modulated pulse train, which enabled sequence lengths of up to 1024 frames [S14]. This modification, however, substantially reduced the imaging speed to 13.3 thousand fps.

*Compressed ultrahigh-speed photography:* Compressed ultrahigh-speed photography systems leverage spatial encoding and temporal shearing to overcome the frame-rate limitations of conventional cameras [15]. Two representative implementations for microbubble imaging are COSDUM and AOFS-CUSI [S16]. The COSDUM system presented in this work achieves a frame rate of 6 Mfps, a sequence depth of 144 frames, and a spatial resolution of 0.52 µm. The AOFS-CUSI system employs frequency-swept LED illumination to capture transient events at rates up to 1.55 Mfps. A broadband LED source is modulated by a time-varying RF signal that drives an acousto-optic tunable filter, producing a sequence of spectrally distinct light pulses. These pulses propagate through the scene, such that each wavelength corresponds to a different temporal frame. Then, a static coded aperture spatially modulates the time-spectrally encoded scene, which is subsequently sheared across different positions on the sensor using a diffraction grating. The encoded signal is recorded using a standard CMOS camera, and up to 31 frames can be computationally reconstructed from a single exposure. The system achieves a temporal resolution of 645 ns and a spatial resolution of 2.2 μm.

## REFERENCES

1. Marquez M, Lai Y, Liu X, Jiang C, Zhang S, Arguello H, et al. Deep-learning supervised snapshot compressive imaging enabled by an end-to-end adaptive neural network. IEEE Journal of Selected Topics in Signal Processing. 2022;16(4):688-699.

2. Gill PE, Robinson DP. A primal-dual augmented Lagrangian. Computational Optimization and Applications. 2012;51(1):1-25.

3. Boyd S, Parikh N, Chu E, Peleato B, Eckstein J. Distributed optimization and statistical learning via the alternating direction method of multipliers. Foundations and Trends® in Machine Learning. 2011;3(1):1-122.

4. Ronneberger O, Fischer P, Brox T, editors. U-net: Convolutional networks for biomedical image segmentation. Medical Image Computing and Computer-Assisted Intervention, proceedings, part III 18; 2015 October 5-9; Munich, Germany: Springer.

5. Zeng T, So HK-H, Lam EY. Computational image speckle suppression using block matching and machine learning. Applied Optics. 2019;58(7):B39-B45.

6. Lebrun M. An analysis and implementation of the BM3D image denoising method. Image Processing On Line. 2012;2:175-213.

7. Deng CY. A generalization of the Sherman–Morrison–Woodbury formula. Applied Mathematics Letters. 2011;24(9):1561-1564.

8. Lauterborn W, Kurz T. Physics of bubble oscillations. Reports on Progress in Physics. 2010;73(10):106501.

9. Yao Y, Liu X, Qi D, Yao J, Jin C, He Y, et al. Capturing transient events in series: a review of framing photography. Laser & Photonics Reviews. 2024;18(12):2400219.

10. Chin CT, Lancée C, Borsboom J, Mastik F, Frijlink ME, de Jong N, et al. Brandaris 128: A digital 25 million frames per second camera with 128 highly sensitive frames. Review of Scientific Instruments. 2003;74(12):5026-5034.

11. Liu X, Kilcullen P, Wang Y, Helfield B, Liang J. Diffraction-gated real-time ultrahigh-speed mapping photography. Optica. 2023;10(9):1223-1230.

12. Liu X, Kilcullen P, Wang Y, Helfield B, Liang J. Ultrahigh-speed schlieren photography via diffraction-gated real-time mapping. Advanced Imaging. 2025;2(1):015001.

13. Kornienko V, Kristensson E, Ehn A, Fourriere A, Berrocal E. Beyond MHz image recordings using LEDs and the FRAME concept. Scientific Reports. 2020;10(1):16650.

14. Kornienko V, Andersson D, Stiti M, Ravelid J, Ek S, Ehn A, et al. Simultaneous multiple time scale imaging for kHz–MHz high-speed accelerometry. Photonics Research. 2022;10(7):1712-1722.

15. Lai Y, Marquez M, Liang J. Tutorial on compressed ultrafast photography. Journal of Biomedical Optics. 2024;29(S1):S11524-S11524.

16. Guo M, He Y, Yao Y, Huang Z, Cheng B, Cao J, et al. Compressive ultrahigh-speed imaging based on acousto-optic frequency sweeping. Photonics Research. 2025;13(10):2967-2975.

## SUPPLEMENTARY FIGURES


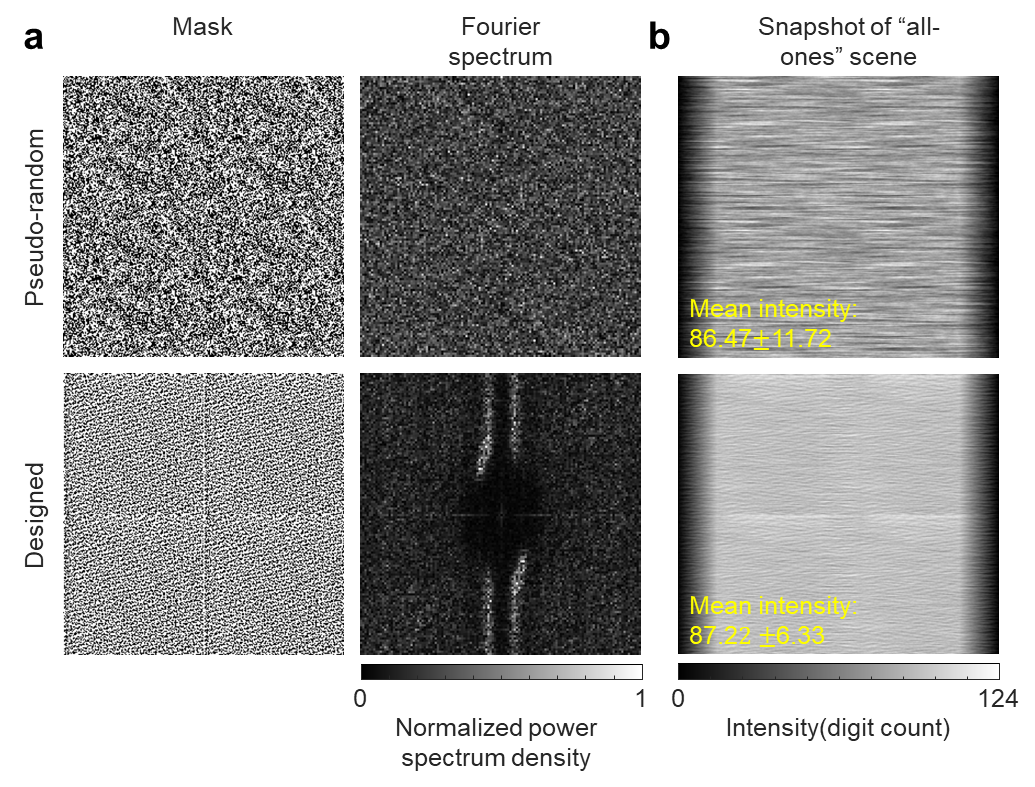


**Fig. S1. Comparison of encoding masks.** a Characteristics of the conventional pseudo-random binary mask and the designed mask obtained via an end-to-end learning approach. b Intensity distributions of COSDUM snapshots (without high-pass filtering**)** for an all-ones scene acquired using the pseudo-random binary mask and the designed mask.


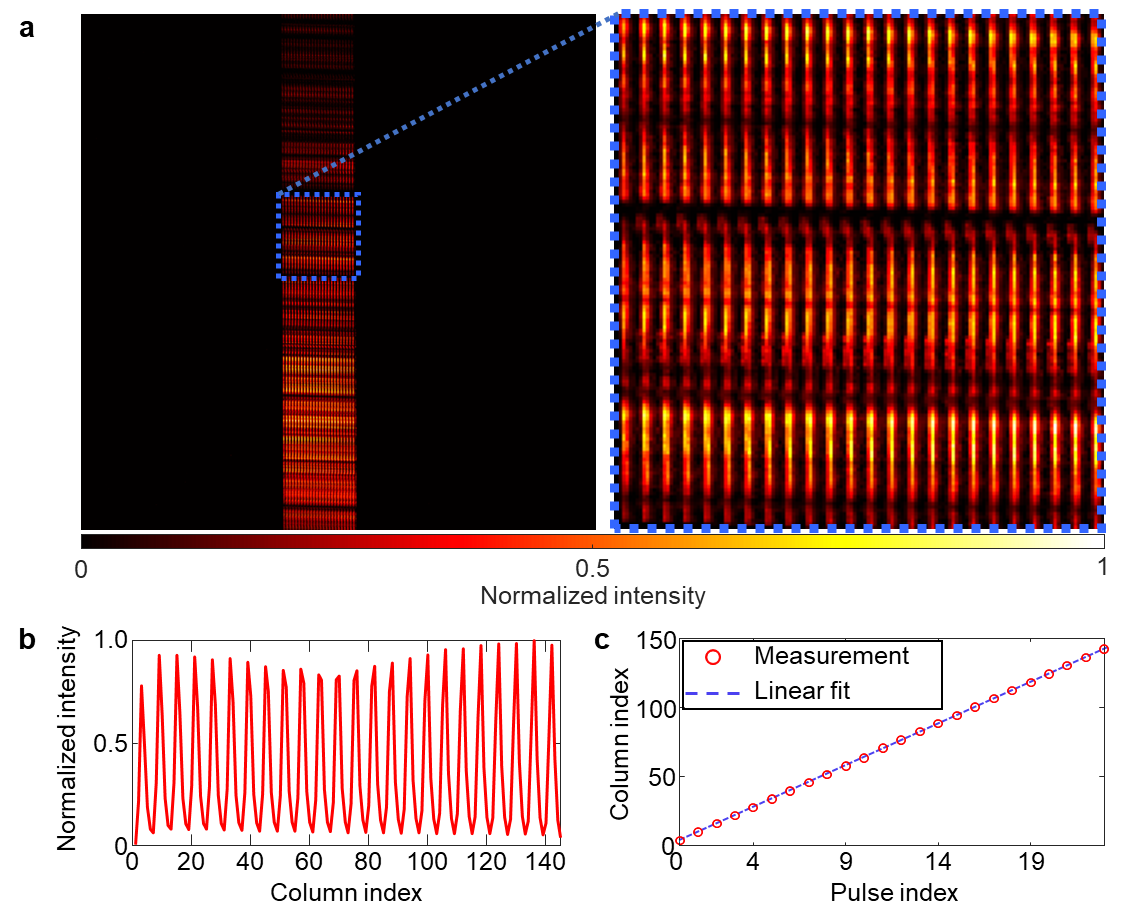


**Fig. S2. Characterization of COSDUM’s imaging speed.** a COSDUM snapshot showing 24 laser pulses acquired over a 24 µs exposure. b Row-averaged intensity. c Linear fit of pulse peak positions.
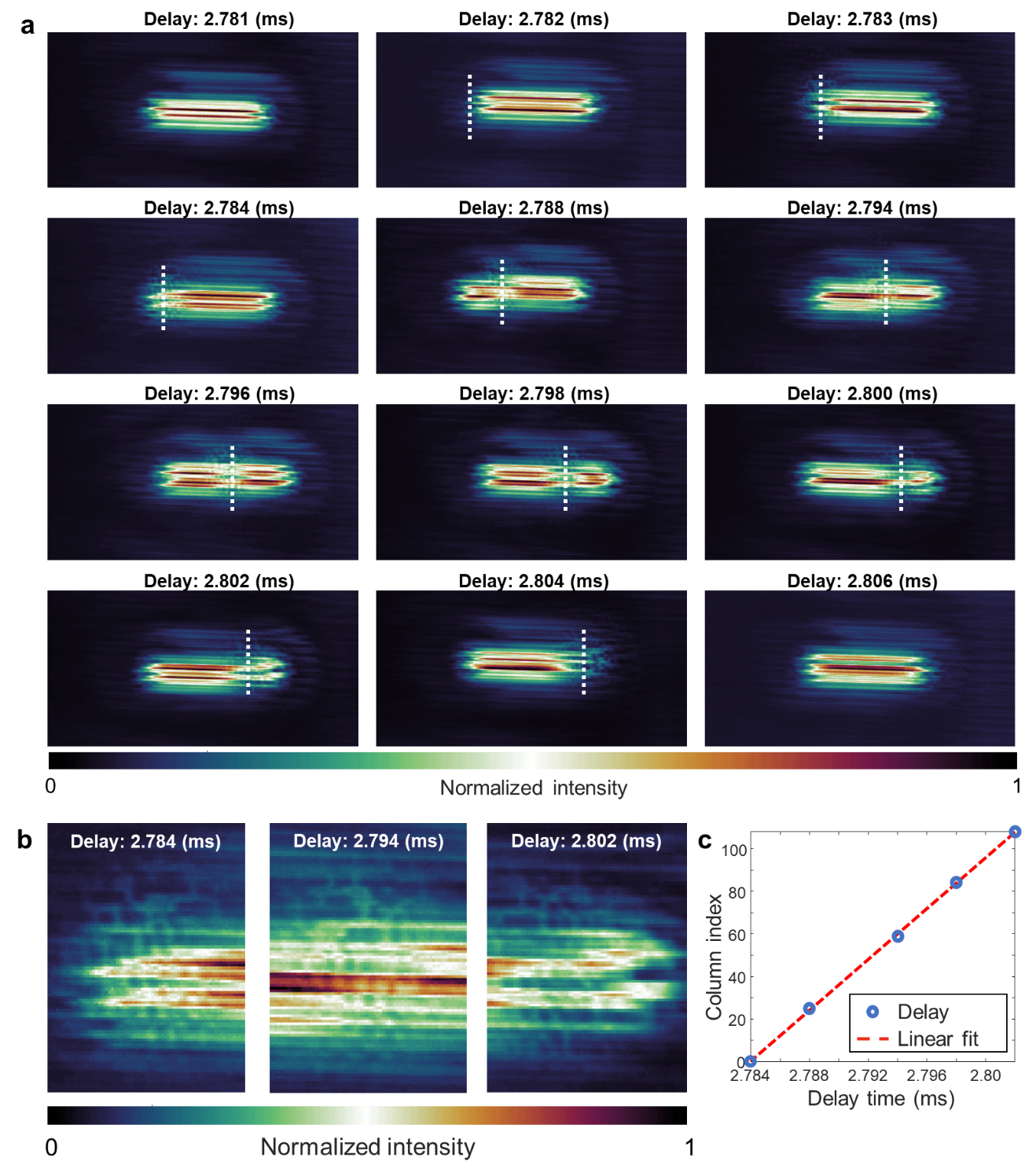


Fig. S3. Time-sheared COSDUM snapshots of a microbubble insonated with a single-cycle, 1 MHz ultrasound pulse. a Sequential frames captured at varying delays, showing the microbubble’s radial dynamics. White dashed lines indicate the relative position of the acoustic pulse as it reaches the microbubble, illustrating the effect of delay tuning on the arrival time of the ultrasound. b Selected frames highlight the spatial shift of the ultrasound wavefront at different delay times. c Column index of the ultrasound wavefront position plotted as a function of delay time.


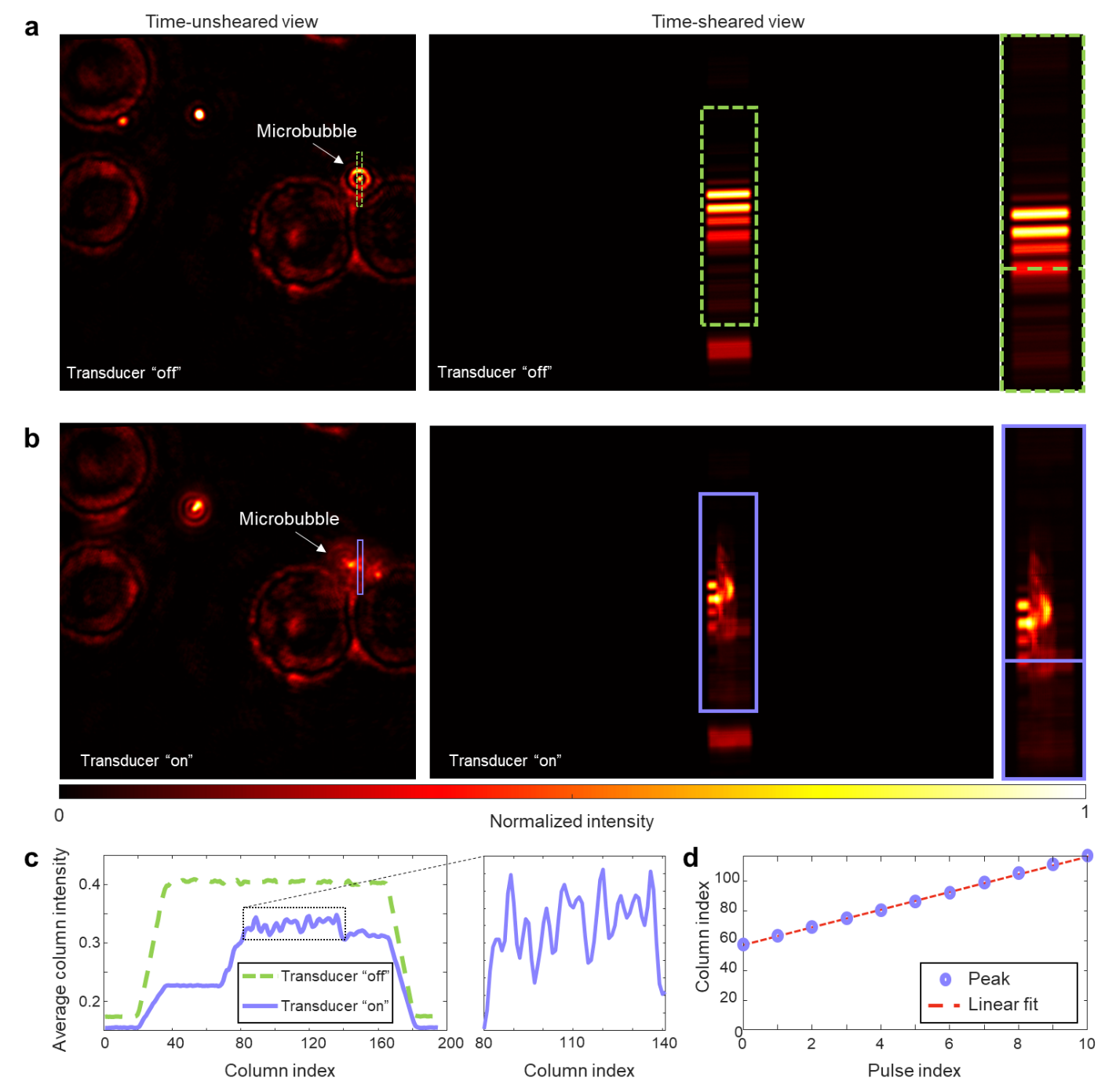


**Fig. S4. Validation of ultrasound synchronization in COSDUM.** **a–b** Snapshots with the ultrasound transducer “off” (a) and “on” (b). A slit replaced the transmissive mask for the acquisition of the time-sheared view. **c** Horizontal line profiles extracted from the time-sheared views (green dashed and purple solid lines), revealing acoustic modulation. A close-up highlights the relative positions of the wavefronts as they reach the microbubble. **d** Linear fit of peak positions.

**
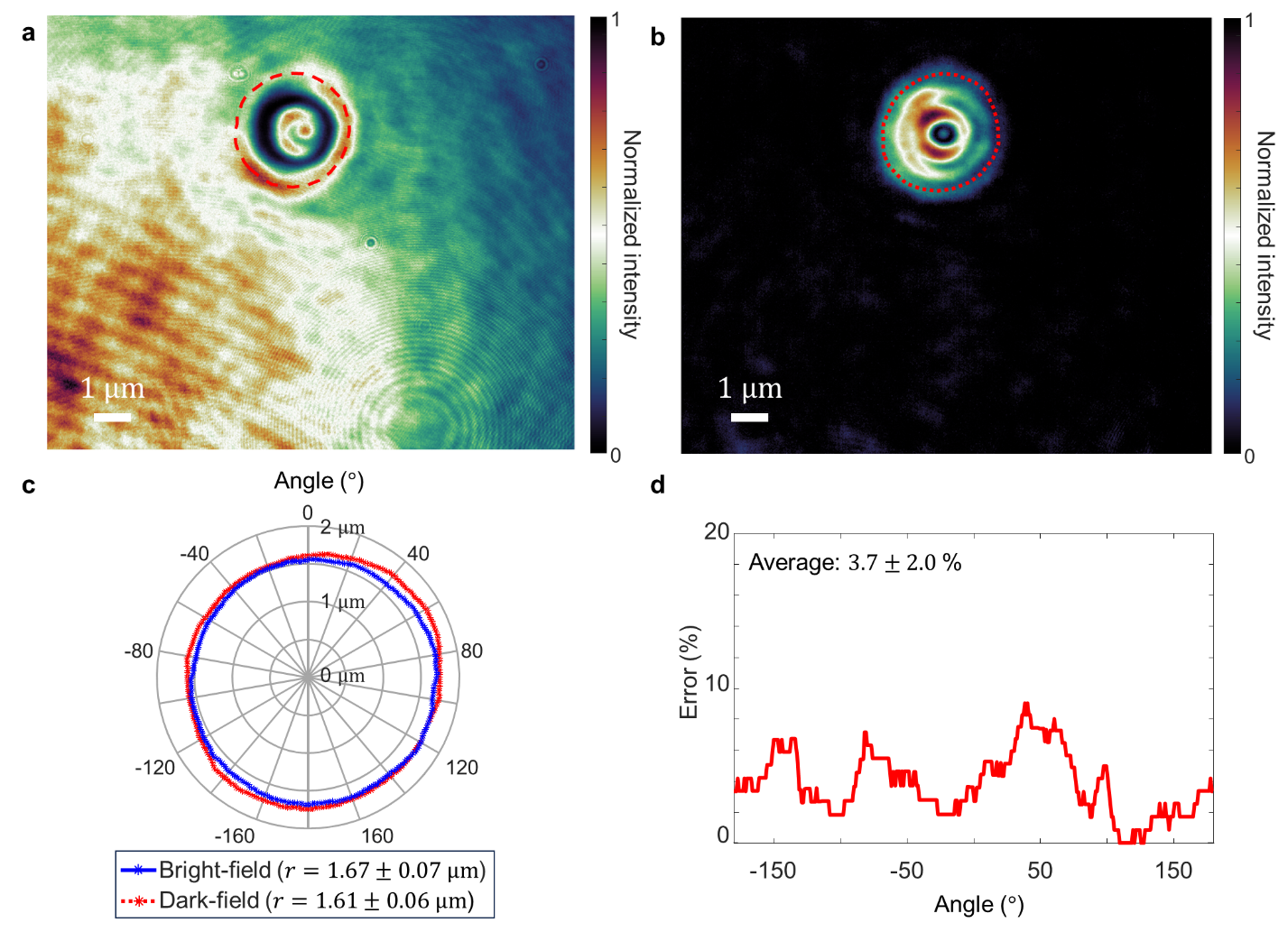
**

**Fig. S5. Microbubble radius analysis using bright-field and dark-field imaging modes.** **a–b** Images of a microbubble captured in bright-field (a) and dark-field (b) imaging mode. **c** Angular radius profiles of the microbubble in the two imaging modes. **d** Percentage error between the bright-field and dark-field radius profiles.


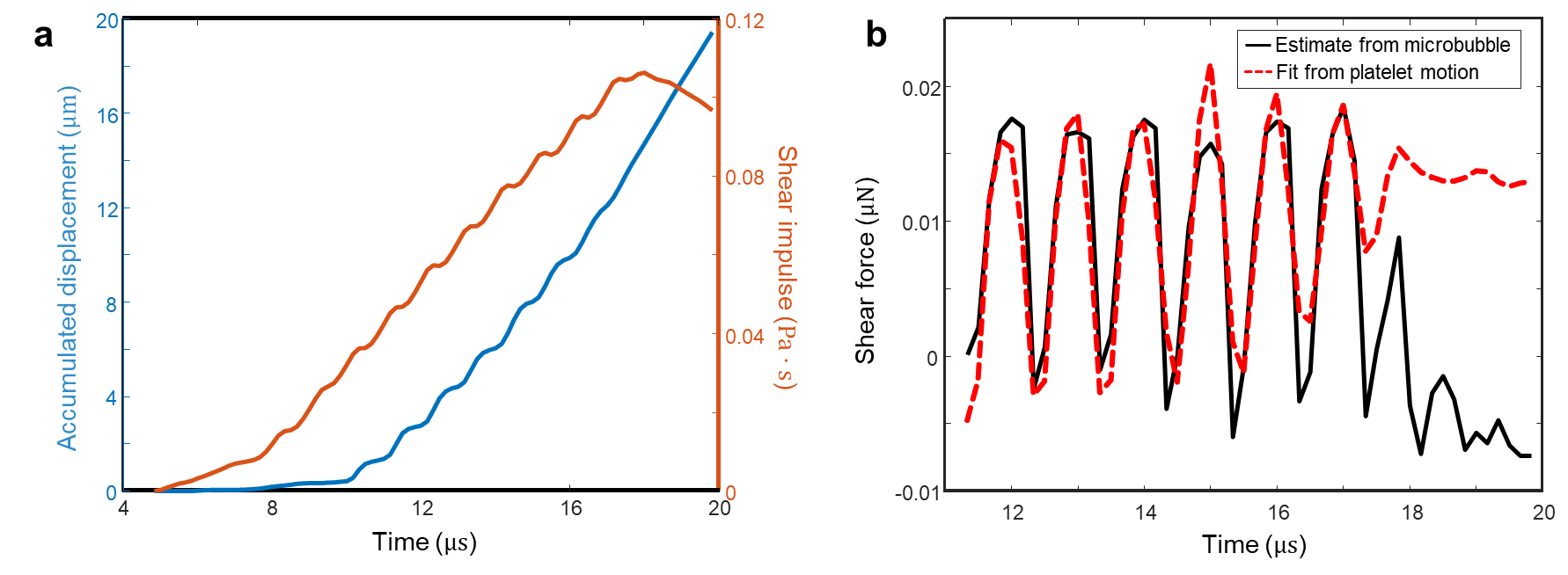


**Fig. S6. Platelet displacement and shear force estimation.** **a** Cumulative platelet displacement and the corresponding shear impulse. **b** Shear force estimated from microbubble dynamics (black solid line) and fitted to platelet motion (red dashed line).

## SUPPLEMENTARY TABLE


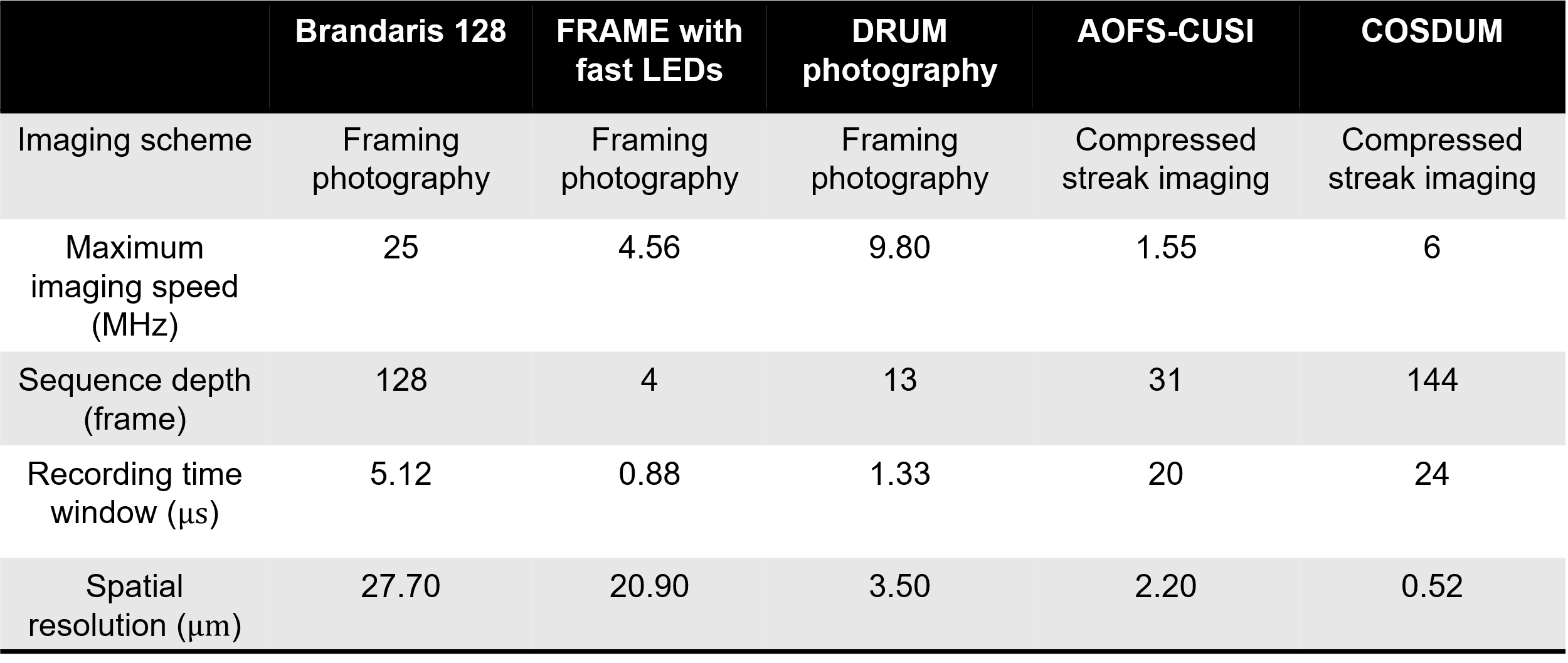


**Table S1. Comparison of representative single-shot imaging systems operating in the million-frame-per-second (Mfps) regime.**

## MOVIE CAPTIONS

**Movie S1.** COSDUM of 50-nm-diameter nanoparticles illuminated by a 1-MHz laser pulse train.

**Movie S2.** COSDUM of stable cavitation in a microbubble insonified using a 10-cycle, 1-MHz ultrasound pulse at a peak negative pressure of 0.3 MPa.

**Movie S3.** COSDUM of the collapse of a microbubble insonated using a 10-cycle, 1-MHz ultrasound pulse at a pressure of 1 MPa.

**Movie S4.** COSDUM of microbubble-platelet interactions in whole blood. The microbubble was insonated with a 10-cycle, 1 MHz ultrasound pulse at 1 MPa.

**Movie S5.** COSDUM of microbubble-red blood cell interaction in whole blood. The microbubble was insonated with a 10-cycle, 1 MHz ultrasound pulse at 1 MPa.
